# Mixing varieties mitigates early root competition in wheat under water and nutrient limitation

**DOI:** 10.1093/jxb/eraf163

**Published:** 2025-04-26

**Authors:** Germain Montazeaud, Pierre Roumet, Mickaël Lamboeuf, Christian Jeudy, Martin Ecarnot, Lise Malicet-Chebbah, Christophe Salon, Hélène Fréville

**Affiliations:** AGAP, Université de Montpellier, CIRAD, INRAE, L’Institut Agro, Montpellier, France; AGAP, Université de Montpellier, CIRAD, INRAE, L’Institut Agro, Montpellier, France; Université de Bourgogne, Agroecol Lab, Université de Bourgogne Franche Comte, AgroSup Dijon, INRAE, Dijon, France; Université de Bourgogne, Agroecol Lab, Université de Bourgogne Franche Comte, AgroSup Dijon, INRAE, Dijon, France; AGAP, Université de Montpellier, CIRAD, INRAE, L’Institut Agro, Montpellier, France; AGAP, Université de Montpellier, CIRAD, INRAE, L’Institut Agro, Montpellier, France; Université de Bourgogne, Agroecol Lab, Université de Bourgogne Franche Comte, AgroSup Dijon, INRAE, Dijon, France; AGAP, Université de Montpellier, CIRAD, INRAE, L’Institut Agro, Montpellier, France; CIMMYT, Mexico

**Keywords:** Agroecology, arms race, competitive hierarchy, high-throughput root phenotyping, niche complementarity, relative yield, root projected area, tragedy of the commons, varietal mixtures, wheat

## Abstract

Competition between plants can lead to a tragedy of the commons (TOC), where excessive investment in resource-harvesting organs reduces collective performance. Mixing crop varieties could resolve such TOCs through niche complementarity—if varieties differ in resource use—or selection effects, where competitive varieties benefit from weaker neighbours. While most studies on varietal mixtures focus on above-ground traits, below-ground interactions remain poorly understood. We grew 36 durum wheat (*Triticum turgidum* ssp. *durum*) varieties in pure stands and 54 binary mixtures using a high-throughput root phenotyping platform, under both non-limiting (R+) and limiting (R–) water and nutrient conditions, to assess early-stage root competition. In R–, mixtures produced less biomass than expected based on pure stands, largely due to a negative complementarity effect. This was mostly explained by the average projected root area of the two varieties. Rather than indicating a negative interaction, the effect reflected a relaxation of competition: varieties with larger root systems benefited from having weaker competitors, disengaging from the arms race for biomass accumulation. These findings suggest that root area is a promising breeding target for mitigating intra-specific competition and a critical trait for assembling optimal varietal mixtures.

## Teaser

Wheat varieties with a high root projected area in pure stands experience a strong relaxation of below-ground competition in mixtures at the seedling stage under resource-limited conditions.

## Introduction

Competition for resources is a fundamental determinant of plant phenotypes and plant community dynamics (Tilman, 2020). Survival, growth, and reproduction of a plant can be reduced by highly competitive neighbours that absorb and use resources more quickly and/or more efficiently. To deal with such situations, plants have evolved various mechanisms to detect the presence of neighbours and/or the early exhaustion of resources, and to react by becoming more efficient at capturing resources or more conservative with their own resources (Pierik *et al.*, 2013). For example, plants can anticipate light competition by detecting changes in the red:far-red ratios of reflected and transmitted light on the leaves of their neighbours (Ballaré *et al.*, 1990). These changes in light quality in turn trigger a series of phenotypic changes, known as the shade avoidance syndrome, which ultimately lead to more vertical growth in order to outcompete neighbours, for example increased plant height or hyponastia of leaves (Ballaré and Pierik, 2017). Similarly, competition for below-ground resources (water or nutrients) can lead to root proliferation in the presence of a competitor (Robinson *et al.*, 1999; Gersani *et al.*, 2001), which can be triggered either by early detection of resource depletion or by the detection of chemical markers from the competitors (Schenk, 2006; Pierik *et al.*, 2013).

While such responses primarily evolved to maximize individual plant fitness under competition, they can lead to a competitive arms race between individuals, and ultimately decrease fitness at the scale of the group, which is known as a tragedy of the commons (TOC; Hardin, 1968). This reduction in collective performance results from two effects: resources allocated to competitive organs are diverted from reproduction, and all individuals suffer from an intense pressure on the resources from their neighbours. Such trajectories have important implications for agriculture, as intense competition between adjacent plants from the same species can reduce yield per unit area (Anten and Vermeulen, 2016). This was notoriously identified by agronomists and plant breeders in the context of the Green Revolution: intra-specific competition, especially for light, becomes a strong determinant of yield in a typical high-density cereal cropping system with low weed pressure and high fertilizer and pesticide inputs (Donald, 1963, 1968; Jennings and de Jesus, 1968). This observation led to the emergence of a new paradigm for plant breeding based on the idea that selection should target varieties with weak competitive abilities, because such varieties can be grown at high density without wasting resources for competition (Donald, 1968). The major yield gains achieved with the introduction of dwarfing genes contributed to validate this paradigm as these genes mainly reduced above-ground plant stature and, *de facto*, the intensity of intra-specific competition (Donald, 1968; Jennings and Herrera, 1968).

Transitioning towards low-input farming practices, notably by reducing the use of fertilizers, will reduce the amount of nutrients readily available for plants and, as such, will exacerbate the relative effect of below-ground compared with above-ground intra-specific competition. When plants compete for soil resources, game theory models predict that individuals will overinvest in root biomass— they will continue allocating biomass in the roots even when the cost of the root system starts outweighing the benefits associated with the increased access to soil resources, leading to a TOC (Hardin, 1968; Zhang *et al.*, 1999; Gersani *et al.*, 2001; Anten and Vermeulen, 2016). Multiple empirical lines of evidence support this prediction: overinvestment in roots in response to competition has been shown to reduce final above-ground biomass in wheat (Zhu *et al.*, 2019) and soybean (Gersani *et al.*, 2001). Topsoil root production also negatively correlates with grain yields in oats and barley (Vain *et al.*, 2023). Thus, resolving below-ground TOCs might be key to maintain high yields under low input farming practices.

Below-ground TOCs could be resolved by conducting direct selection on root architectural and morphological traits, for example by selecting smaller root systems with narrower angles as proposed by Donald (1968). Direct selection on the size of resource-foraging organs in the above-ground compartment was very efficient to increase yield in the past, as exemplified with dwarfing genes. However, because they are much less visible, root traits have received considerably less attention (Anten and Vermeulen, 2016). Even with modern phenotyping tools, direct selection on root traits might be challenging and costly, and we only have little information on how these traits respond to selection (Kuijken *et al.*, 2015). Alternatively, one could use the principles that prevent the evolution of TOCs in natural ecosystems, notably group selection and kin selection, and apply them in plant breeding to select crops that have less competitive (or more cooperative) root systems, which is the core idea of Darwinian Agriculture and Evolutionary Agroecology (Denison *et al.*, 2003; Weiner *et al.*, 2017). Theoretical models suggest that these evolutionary principles can be used to avoid TOCs and to select more cooperative crops (Montazeaud *et al.*, 2020; Biernaskie, 2022). However, they are not straightforward to implement in practice, and they rely on high genetic relatedness between individuals, meaning that they produce varieties in which individuals are genetically homogeneous. Both ecological and agronomic research, in contrast, suggest that there are multiple benefits to increasing genetic diversity within crop stands. For example, mixing different varieties in the same field can be very efficient at limiting pathogen spreads and disease severity (Wolfe, 1985; Mundt *et al.*, 1995; Zhu *et al.*, 2000; Finckh and Wolfe, 2006), and, on average, varietal mixtures yield 2–5% more than expected on the basis of their pure stand components, which is known as overyielding (Kiær *et al*., 2009; Borg *et al.*, 2018; Reiss and Drinkwater, 2018; Beillouin *et al*., 2021).

Reducing intra-specific competition for resources while increasing genetic diversity in the field could thus be the optimal way to take advantage of intra-specific plant–plant interactions in agriculture. Two ecological mechanisms are known to affect competition in diversified plant communities: the niche complementarity effect and the selection effect (Loreau and Hector, 2001). Both effects can be either positive or negative in sign. The niche complementarity effect results from niche segregation between species: because different species use different resources (or use the same resources differently, e.g. at different times), the species grown together are more efficient at using the global pool of resources than the individual species in pure stands. In that case, the niche complementarity effect is positive: on average, all species benefit from reduced competition when grown with an inter-specific neighbour (MacArthur and Levins, 1967). In natural plant communities, rooting depth differences between species are believed to be a major driver of below-ground positive complementarity effects (Parrish and Bazzaz, 1976; Mueller *et al.*, 2013). While rooting depth differences have never been associated with complementarity effects in varietal mixtures (Montazeaud *et al.*, 2018), complementarity spatial root distribution can improve water and nutrient uptake in more complex crop assemblages that associate species with contrasted root architectures (Postma and Lynch, 2012; Schmutz and Schöb, 2023). In some cases, complementarity effects can also be negative, meaning that all the species produce less biomass in the mixture than they do in pure stands. This can be caused by physical or chemical interferences between the species, such as when some species attract the pathogens from other species (Wardle *et al.*, 1998; Loreau and Hector, 2001; Polley *et al.*, 2003). Selection effects favour species with specific traits in the mixture (Loreau and Hector, 2001). For example, competitive species can benefit from relaxed competition in a mixture because the mixture allows them to escape an arms race with themselves. If the favoured species are the most productive in pure stands, then the selection effect is positive and it can contribute to overyielding. Such an effect has, for example, been reported in tree communities (Schmid and Niklaus, 2017; Williams *et al.*, 2017) and in inter-specific crop mixtures (Li *et al.*, 2018). Selection effects can be driven by below-ground traits conferring dominance to particular plant types; for example, in grassland communities grown under dry conditions, overyielding is driven by the dominance of deep-rooting species (Bakker *et al.*, 2019). Selection effects can also be negative (i.e. decreasing overyielding) when the species that are favoured in the mixtures are the least productive (Loreau and Hector, 2001; Polley *et al.*, 2003).

Below-ground TOCs could thus be resolved by increasing plant diversity in the field, which could promote ecological effects such as positive complementarity and selection effects. In natural plant communities, these effects are heavily dependent on the level of resources available for the plants. For example, positive complementarity effects are on average stronger under low nutrient conditions in grasslands (Craven *et al.*, 2016), which aligns with the more general observation that positive plant–plant interactions tend to be stronger under harsher environments (i.e. the stress-gradient hypothesis; Bertness and Callaway, 1994; Maestre *et al.*, 2009). These observations suggest that varietal mixtures could hold interesting promise for agriculture, providing adaptations not only to input reductions but also to environmental stresses such as drought. However, we still know very little about the ecological mechanisms at play in varietal mixtures, especially below-ground, and how they are affected by resource availability (Borg *et al.*, 2018).

In the present study, we investigated the potential of varietal mixtures to reduce intra-specific below-ground competition at the seedling stage in durum wheat (*Triticum turgidum* ssp*. durum*). We used a panel of 36 varieties previously shown to exhibit contrasted responses to combined water and nitrogen limitation, and grew them in monogenotypic pots and binary mixtures under highly controlled climate condition in a high-throughput root phenotyping platform under both non-limiting and limiting water and nutrient conditions. We hypothesized that (i) root responses to neighbours are already expressed at the seedling stage; (ii) such responses are more important under resource-limited conditions; (iii) overinvestment in biomass at the seedling stage reflects a response to neighbour competition; and (iv) varietal mixtures can mitigate such responses through either complementarity or selection effects.

## Materials and methods

### Plant material

This study made use of field data collected on a diversity panel of 250 durum wheat genotypes, which was assembled during the EU Project SolACE (https://www.solace-eu.net/) to analyse wheat responses to combined water and nitrogen limitations (Collet, 2022). This panel comprised genotypes from four different collections (Collet, 2022): (i) ‘CREA’, with Italian cultivars, worldwide cultivars, and breeding lines selected by CREA (Consiglio per la Ricercar in agricoltura e l’analisi dell’Economia Agraria, Italy); (ii) ‘EPO’, a highly diverse collection of fixed lines derived from an evolutionary pre-breeding population (David *et al*., 2014); (iii) ‘GPDUR’, with old and modern cultivars from various geographic areas including Western Europe; (iv) ‘UNIBO’, a diversity panel comprising genotypes from pre-breeding programmes, elite lines, and representative genotypes of several worldwide breeding programmes since the 1970s. Based on both field and platform data, a subset of 36 genotypes exhibiting contrasted yield responses to resource availability (i.e. ranging from stable to unstable yields) was selected by the European consortium to perform further experiments. Information on the 36 genotypes can be found in Supplementary Table S1.

### Experimental design

**Fig. 1.**
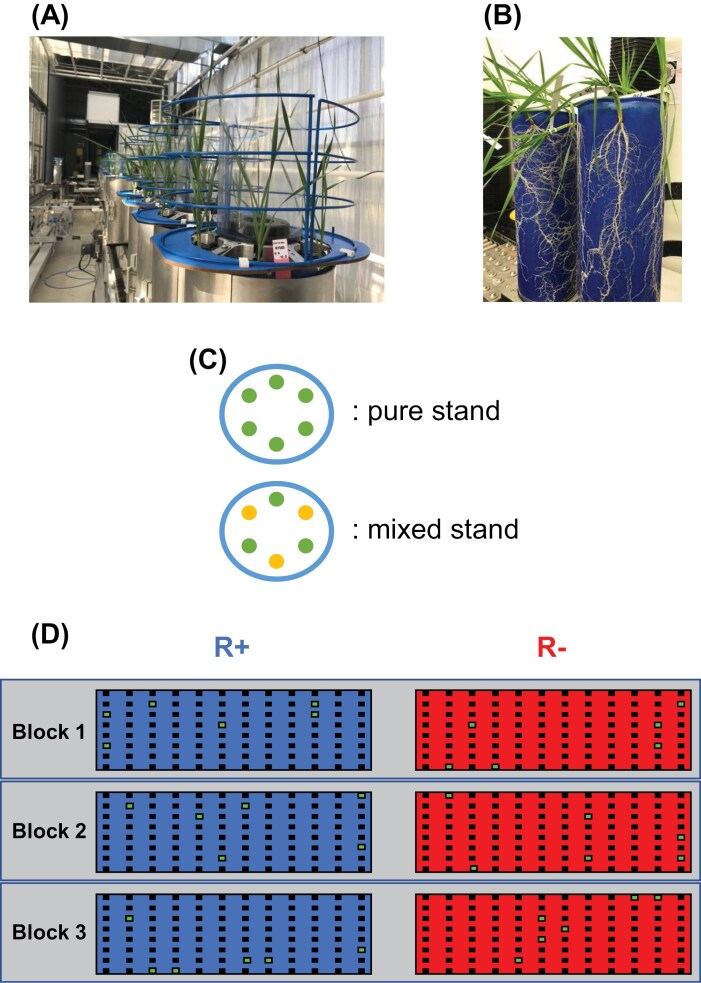
Experimental design. (A and B) Close-up views of RhizoTubes® showing wheat seedlings and their roots. (C) Spatial distribution of the seedlings within the RhizoTubes® in both pure (up) and mixed (bottom) stands. Different colours represent different genotypes. (D) Schematic representation of the experiment; each dark square represents a RhizoTube®. The green squares are the control RhizoTubes® and are all grown with the same wheat variety to check environmental heterogeneity.

Based on the 36 genotypes, we designed a set of 54 binary mixtures selected at random, with each genotype observed in three different mixtures (Supplementary Table S2). Pure stands (here referring to groups of a single genotype, *n*=36) and mixtures (here referring to groups of two genotypes, *n*=54) were grown in RhizoTubes® (Jeudy *et al.*, 2016), transparent pots designed for high-throughput root phenotyping (Fig. 1A, B), with six plants per RhizoTube® placed ~9 cm apart. The six plants had the same genotype in pure stands, whereas two genotypes were grown in alternate positions in the mixtures (Fig. 1C). The 36 pure stands and the 54 mixtures were grown under unlimited resource conditions (R+ treatment) or under combined water and nutrient limitation (R– treatment). All pure stands and mixtures were replicated three times within each treatment following a randomized complete block design within treatment, leading to a total of 540 RhizoTubes® (Fig. 1C). In addition, six RhizoTubes® per experimental block per treatment (i.e. 36 RhizoTubes® in total) were grown with the commercial variety ANVERGUR and were used as controls to check for environmental heterogeneity in the greenhouse.

### Growth conditions

Seeds were first disinfected with a solution of 6 g l^–1^ active chlorine (four tablets per litre of a standard commercial chlorine) in which they were immersed, agitated for 15 min, and finally rinsed 10 times with sterile water on 22 June 2019. They were then soaked overnight, and sown into Petri dishes on 23 June. They stayed at 4 °C for 24 h before being transplanted into the RhizoTubes® on 24 June. RhizoTubes® have a diameter of 17 cm and a depth of 49.5 cm (Jeudy *et al.*, 2016). They were filled with a 25:75 mixture of sand (Biot B4, Silices et Refractaires de la Méditerranée) and perlite. Temperatures were maintained at ~20–25 °C, relative humidity at ~70–80%, and photoperiod was set to 16 h, with an average photosynthetically active radiation (PAR) of 330 µmol m^–2^ s^–1^ during the day. Seedlings were provided daily with a liquid nutrient solution that contained water, N, P, K, and all micronutrients required for plant growth (Supplementary Table S3). The water content of each RhizoTube® was monitored each day, and the amounts of nutrient solution were adjusted to maintain the RhizoTubes® at 100% of their water storage capacity (Supplementary Fig. S1). In the R– treatment, the provision of nutritive solution was stopped on 28 June (4 d after seedling transfer), causing the water content to decrease, ultimately reaching 55% of the full storage capacity by the end of the experiment (Supplementary Fig. S1). Given the size of the experiment (3240 plants to be phenotyped at harvest), the plants were harvested on four consecutives dates, between 16 and 19 July (i.e. ~3 weeks after transplantation). All were at the beginning of the tillering stage.

### Phenotyping

Root traits were measured for each RhizoTube® based on image analysis as described in Jeudy *et al.* (2016). Briefly, the root detection is based on a custom image segmentation process that extracts the root pixels (white or near-transparent) from the contrasted bluish background. Thresholding on the red channel of the RGB image and phase preservation in the frequency domain are used to obtain the most contrasted image possible. A few morphological operations are then applied to clean up artefacts and refine the detection of root borders. Finally, light pixels are set to white, representing the roots, while the remaining pixels are set to black to indicate the background. The architecture of the root system is then characterized by locating and counting the white pixels in two dimensions, and converting distances and dimensions into centimetres or millimetres using the image resolution. We used images taken on 15 July—as close as possible to harvest—in order for them to have sufficiently developed root systems and to synchronize root trait measurements and biomass measurements as much as possible in order to increase the chance of detecting causal relationships between them. On this date, however, the roots of the different plants were overlapping in most of the RhizoTubes®, and the image processing algorithm was not able to isolate the root systems of individual plants. We thus aggregated root traits at the level of the RhizoTube® as a whole. Three root traits could be computed following this aggregation: root depth, corresponding to the distance between the top of the RhizoTube® and the deepest root pixel; root length, the total length of roots detected on the image; and root area, the 2D projected area of the total root system. Root area combines information on both root length and root diameter. We did not consider root depth in our analyses because root tips reached the bottom of the RhizoTubes® in most cases, leading to highly left-skewed trait distribution and very low trait variability.

At harvest, we extracted plants from the RhizoTubes®. Because the above-ground organs were clearly separated between the neighbouring plants, we could separate individual plants by carefully and manually disentangling their root systems. Then, contrary to root traits measured via image analysis, we were able to measure above-ground and biomass traits at the level of individual plants. For each plant, we counted the number of leaves on the main tiller (hereafter ‘# leaves’) and the total number of tillers (hereafter ‘# tillers’). Above- and below-ground biomass were then separated for each plant and dried before weighing to determine shoot biomass, root biomass, root:shoot ratio, and total biomass. Leaf nitrogen content (hereafter ‘leaf N’) was measured with near-infrared spectrometry (NIRS). We measured one NIR spectrum per leaf per plant in each RhizoTube® (i.e. six spectra per RhizoTube®) using the Fieldspec 2500© (Analytical Spectral Devices, Inc., Boulder, CO, USA) spectrometer. NIRS measurements were done 1 d before the harvest (i.e. on 15 July for the RhizoTubes® harvested on 16 July, on 16 July for the RhizoTubes® harvested on 17 July, etc.). NIR spectra were converted into nitrogen content using the calibration described in Ecarnot *et al.* (2013).

### Statistical analysis

We performed all statistical analyses with R v. 4.3.2 (R Core Team, 2019).

We first tested the effect of the treatment (R+ versus R–) on the different traits and biomass components (above- and below-ground) using only pure stand data summed per RhizoTube® (except leaf N which was averaged per RhizoTube®). We used mixed linear models with a given trait or biomass component as the response variable, treatment as a fixed effect, and genotype identity as a random intercept and random treatment slope. We also included two covariates as fixed effects: block, and harvest date or measurement date (except for root traits which were all measured on the same day). We assessed the significance of the fixed effects with standard ANOVA and *F*-statistics computed with Kenward-Roger’s approximations for the degrees of freedom (Supplementary Table S4). We fitted the mixed model with the lmer() function (package lme4), and checked significance with the anova() function (package lmerTest).

To compare the relative biomass of mixed versus pure stands, we computed the relative yields (RYs; de Wit and van den Bergh, 1965) of the varieties in mixed stands for each biomass component (above-ground, below-ground, and total biomass):


$$\begin{array}{l} RY_{ijk}=\frac{B_{ijk}}{B_{iik}}, \end{array}$$ (1)

Where RY_*ijk*_ is the relative yield of the variety *i* grown in mixture with the variety *j* in the treatment *k*, B_*ijk*_ is the biomass (above-ground, below-ground, or total biomass) of the variety *i* grown in mixture with the variety *j* in the treatment *k*, and and B_*iik*_ is the reference biomass of pure stands of the variety *i* in treatment *k*. As described above, we harvested the experiment on four consecutive days. To avoid confounding effects between harvest date and other factors, notably treatment (R+ versus R–) and spatial blocks, we harvested a quarter of each block within each treatment each day (Fig. 1D). This means that, within each block×treatment combination, the RhizoTubes® had four different harvest dates. As a consequence, it was not possible to compute RY and compare pure and mixed stands within treatment×block combinations, because—for a given mixture—the harvest date could be different between the mixture and the two pure stand components. Instead, we first separated our dataset between the pure and mixed stands. Then, we summed the biomass of all plants of the same genotype within each RhizoTube® (i.e. six plants in pure stands, and three plants in mixed stands). We used the dataset of the pure stands to compute the reference biomass of pure stands. To do so, we fitted a linear mixed model with biomass as the response variable, harvest date, block, and treatment as fixed effects, and variety identity as a random effect (here we included only the random effect of the variety on the intercept, as adding the random effect on the treatment slope led to singular models due to very low variance on the slope). We then summed the best linear unbiased predictor (BLUP) of each variety with the estimated fixed effect of the treatment (R+ or R–) to obtain the reference biomass values of pure stands adjusted for the effects of block and harvest date within each treatment (i.e. B_*iik*_). For mixed stands, we also fitted a linear mixed model with each variety biomass as the response variable, harvest date, block, and treatment as fixed effects, and variety pair identity as a random effect on the intercept and on the treatment slope. Pair identity was constructed as the concatenation of the identity of the focal and neighbour variety, such that variety 1 and variety 2 in a mixed stand had pair identities variety1–variety2, and variety2–variety1, respectively. This allowed us to obtain one BLUP value for each variety within each variety combination, whereas using the same pair identity for both components of the mixture (e.g. variety1–variety2 for both) would have yielded a single value for the two components. We then summed the BLUP value of each variety (intercept in the R+ treatment, intercept+slope in the R– treatment) with the estimated fixed effect of the treatment (R+ or R–) to obtain one biomass value for each variety within each variety combination that was adjusted for the effects of the block and harvest dates within each treatment (i.e. B_*ijk*_). We then divided these mixed stand values by the reference values of the pure stands to obtain RYs for each variety within each mixture following Equation 1. Under the null hypothesis that the variety produced an equal amount of biomass in mixed and in pure stands, RY=0.5 because there are half the number of plants of a variety in mixed stands (*n*=3) compared with pure stands (*n*=6). RY >0.5 means that the variety produced more biomass in mixed than in pure stands, and RY <0.5 means that the variety produced less biomass in mixed than in pure stands.

In a second step, we summed the RYs of the two varieties in the mixed stand to obtain the relative yield total (RYT; de Wit and van den Bergh 1965) of the mixture:


$$\begin{array}{l} RYT_{\left(ij\right)k}=\;RY_{ijk}+RY_{jik}, \end{array}$$ (2)

where RYT_(*ij*)*k*_ is the relative yield total of the mixture containing varieties *i* and *j* in treatment *k*. RYT=1 means that the mixture as a whole produced a similar amount of biomass to that expected from the productivity of the varieties grown in pure stands, whereas RYT >1 means that the mixture produced more biomass than expected, and RYT <1 means that the mixture produced less biomass than expected.

For each mixture within each treatment, we then computed complementarity and selection effects (CE and SE, respectively) following the additive partitioning proposed by Loreau and Hector (2001):


$$\begin{array}{llll} NBE & =Y_{o}-Y_{E}=CE+SE,with\;CE\; & =N\bar{\Delta RY}\;\bar{M}\;and\;SE\; & =Ncov\left(\Delta RY,\;M\right), \end{array}$$

Where NBE is the net biodiversity effect (highly correlated to RYT), *Y*_o_ is the observed biomass of the mixture, *Y*_E_ is the expected biomass of the mixture based on the pure stand biomasses and mixture proportions, *N* is the number of components in the mixture (here *N*=2), ΔRY is the difference between the observed RY and the expected RY of the mixture (i.e. simply its proportion seeded, here expected RY=0.5), and *M* is the pure stand biomass. We computed CE and SE using the apm() function from the bef package (https://github.com/BenjaminDelory/bef).

We then compared RYT, CE, and SE between the R+ and R– treatments using a linear mixed model with RYT, CE, or SE as the response variable, treatment as a fixed effect, and varietal mixture identity (i.e. non-oriented concatenation of the names of the two varieties grown in mixture) as a random effect on the intercept. We assessed the significance of the fixed effects as detailed above (Supplementary Tables S5, S6). Finally, we checked whether RYTs significantly differed from 1 and CE and SE from 0 within each treatment using two-sided *t*-tests.

To assess whether the trait composition (above- and below-ground traits) of the mixture affected varietal interactions, we used traits measured in pure stands to predict the RYT, CE, and SE of mixed stands. This approach is based on the hypothesis that pure stand traits are the information available to agronomists and plant breeders when designing varietal mixtures. First, we computed reference trait values for pure stands that we corrected for design effects and measurement dates. We applied the same methodology for # leaves, # tillers, leaf N, root length, and root projected area. As described above, we fitted a linear mixed model with pure stand trait as the response variable, block, measurement date, and treatment as fixed effects, and varietal identity as a random effect. Varietal identity random effect was specified both on the intercept and on the treatment slope, except for # leaves where adding the random slope led to singular models due to very low variance on the slope. We then computed reference trait values of pure stands as the sum of the BLUP of each variety (intercept in the R+ treatment, intercept+slope in the R– treatment when random slope was specified) with the estimated fixed effect of the treatment (R+ or R–). Then, for each mixed stand, we computed both the average and the absolute trait difference between the two varieties using the reference trait values of the pure stands. Finally, we fitted a linear model with RYT, CE, or SE as the dependent variable and all trait averages and all trait differences as independent variables [lm() function from the stats package]. We standardized all dependent and independent variables (μ=0, σ=1) and ran a backward model selection [glmulti() function from the glmulti package]. We used the second-order Akaike information criterion (AIC_C_; Sugiura 1978) to rank the models and performed model-averaging inference based on the top 10 models using the coef() function (glmulti package). We report parameter estimates and their 95% unconditional confidence interval computed as ±1.96 unconditional sampling SD, variable importance, and adjusted *R*^2^ averaged over the top 10 models (Burnham and Anderson, 2002). Detailed information on the top 10 models is available in Supplementary Tables S7 and S8.

Finally, to further investigate the ecological mechanisms behind the significant relationships between RYT and trait composition, specifically root area, we assessed the relationship between pure stand biomass and pure stand root area, and between RYs and pure stand root area using simple linear regressions between these variables within each treatment. We used trait and biomass values adjusted for block effects and measurement date effects as explained above, such that we had one value per variety per treatment. We also checked the relationship between RYs and the hierarchical distance in root area between the variety and its neighbour in pure stands. Hierarchical distance was the difference between the focal root area and the neighbour root area, divided by the focal root area (i.e. positive values mean that the focal root had a higher area that its neighbour, and vice versa). Finally, we tested whether root area plasticity affected RYT in the two treatments. We computed root area plasticity as the difference between the average root area of the pure stands of the two varieties and the observed root area of their mixture, divided by the average of the pure stands. (i.e. positive values mean that root area increased in the mixed relative to the pure stands, and vice versa).

## Results

### Effect of resource availability on plant growth and traits

**Fig. 2.**
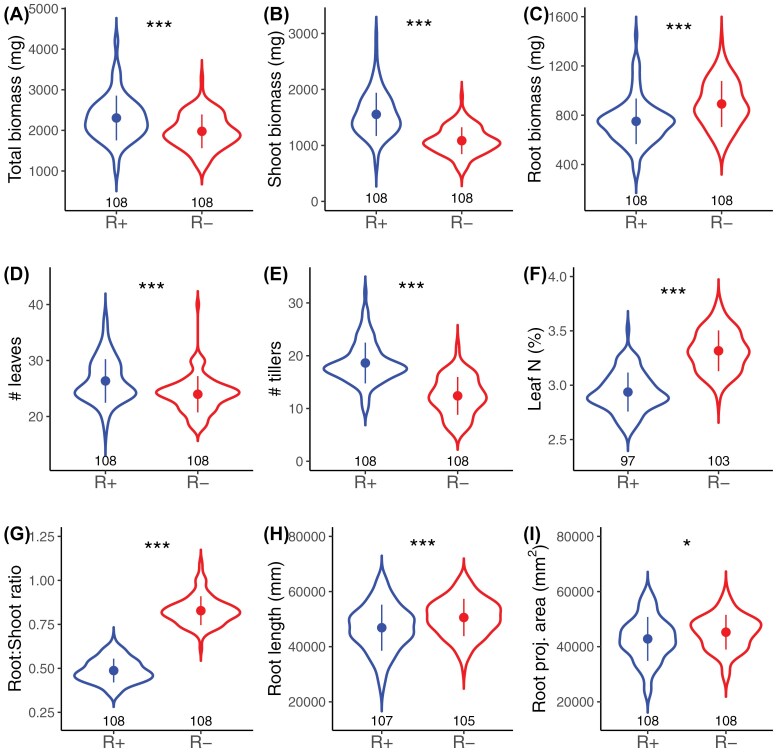
Effect of resource limitation on plant growth and traits. Comparison of total biomass (A), shoot biomass (B), root biomass (C), number of leaves (D), number of tillers (E), leaf nitrogen concentration (F), root:shoot ratio (G), root length (H), and root projected area (I) between the R+ (blue) and the R– (red) treatments. Only pure stand data were used, and trait values were summed per RhizoTube® (except for leaf N for which we averaged trait values per RhizoTube®). Points and error bars represent the mean ±SD. The number of observations in each treatment is reported below each violin plot. Symbols above the plots represent the significance of the treatment effect (*P<0.05, **P<0.01, ***P<0.001; complete ANOVA is reported in Supplementary Table S4).

The limitation of water and nutrients strongly reduced total plant biomass in pure stands (1947.35 mg in the R– treatment versus 2364.71 mg in the R+ treatment, *F*_1,87.77_=144.49, *P*<0.001, Fig. 2A). Such biomass reduction was accompanied by a reallocation of resources from the above-ground to the below-ground compartment: while plants produced less above-ground biomass (Fig. 2B), fewer leaves (Fig. 2D), and fewer tillers (Fig. 2E), they produced higher root biomass (Fig. 2C) and longer roots (Fig. 2H), and had higher root areas (Fig. 2I) in the R– treatment. Consequently, the root:shoot ratio increased from 0.48 in the R+ treatment to 0.82 in the R– treatment (+71%, *F*_1,87.78_=3966.58, *P*<0.001, Fig. 2G). Leaf nitrogen concentration also increased in the R– treatment (3.32% versus 2.95%, *F*_1,87.19_=525.03, *P*<0.001, Fig. 2F).

### Relative biomass of mixtures

**Fig. 3.**
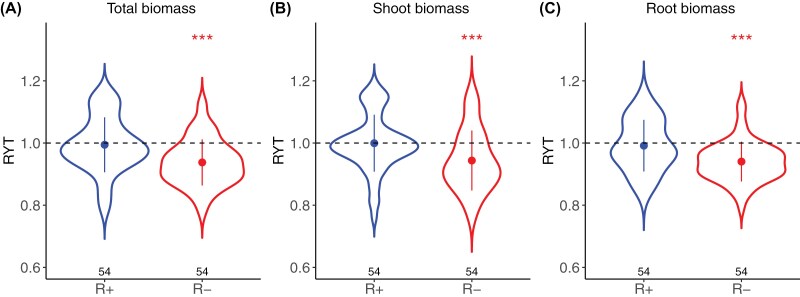
Effect of resource limitation on interactions between varieties. Comparison of relative yield total (RYT) indices on total biomass (A), shoot biomass (B), and root biomass (C) between the R+ (blue) and the R– (red) treatments. Points and error bars represent the mean ±SD. The number of observations in each treatment is reported below each violin plot. Symbols above violins represent the significance of a two-sided *t*-test testing if the mean RYT within treatment is significantly different from 1 (***P<0.001). ANOVAs testing the significance of treatment effect on RYTs are reported in Supplementary Table S5.

RYTs were significantly different between the R+ and R– treatments for all biomass components (Fig. 3; Supplementary Table S5). In the R+ treatment, the biomass production of the mixtures did not significantly differ from the biomass expected from their pure stand components (average RYT for total biomass=0.99, *t*_53_= –0.46, *P*=0.0.6473, Fig. 3A; average RYT for shoot biomass=1.00, *t*_53_= –0.05, *P*=0.9632, Fig. 3B; average RYT for root biomass=0.99, *t*_53_= –0.75, *P*=0.4568, Fig. 3C). In contrast, in the R– treatment, mixtures produced significantly less biomass than expected from the biomass of their components grown in pure stands (average RYT for total biomass=0.92, *t*_53_= –6.18, *P*<0.001, Fig. 3A; average RYT for shoot biomass=0.94, *t*_53_= –4.29, *P*<0.001, Fig. 3B; average RYT for root biomass=0.94, *t*_53_= –6.83, *P*<0.001, Fig. 3C).

### Ecological effects underlying the relative biomass of the mixtures

**Fig. 4.**
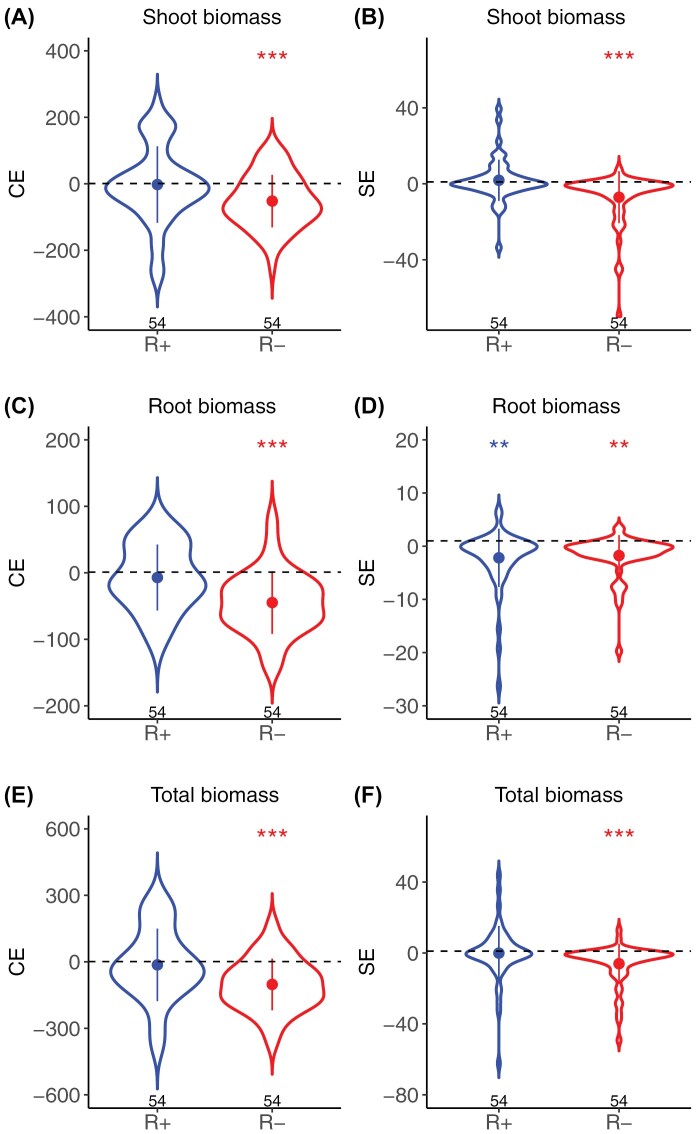
Effect of resource limitation on complementarity and selection effects. Comparison of CEs and SEs on shoot biomass (A, B), root biomass (C, D), and total biomass (E, F) between the R+ (blue) and the R– (red) treatments. Points and error bars represent the mean ±SD. The number of observations in each treatment is reported below each violin plot. Symbols above violins represent the significance of a two-sided *t*-test testing if the mean biodiversity effect within treatment is significantly different from 0 (***P<0.001, **P<0.01). ANOVAs testing the significance of treatment effect on biodiversity effects are reported in Supplementary Table S6.

Complementarity and selection effects were both significantly smaller in the R– than in the R+ treatment for all biomass components, except selection effects on root biomass that were not significantly different between the two treatments (Supplementary Table S6). In the R+ treatment, complementarity and selection effects were not significantly different from zero for above-ground biomass and total biomass, whereas the selection effect was significantly negative for root biomass (Fig. 4). In contrast, in the R– treatment, both complementarity and selection effects were significantly negative for all biomass components (Fig. 4). Complementarity effects were overall stronger than selection effects (e.g. for total biomass, mean complementarity effect= –57.90±148.35 mg, mean selection effect= –3.80±15.76 mg (mean ±SD).

### Effect of trait composition on mixture biomass

**Fig. 5.**
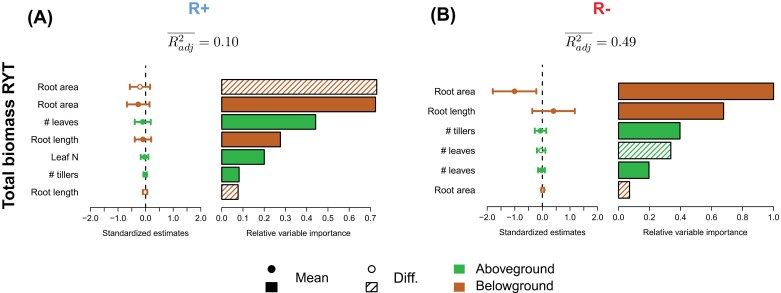
Relationships between the trait composition of the mixtures and their performance. Standardized effects of traits on mixture RYTs measured on total biomass in the R+ treatment (A) and R– treatment (B). Backward model selection was performed on a full model with RYT as the response variable and all trait means and differences as explanatory variables. Based on AIC_C_, the top 10 models were retained to compute model-averaged estimates reported on the left side of the panels with their 95% unconditional confidence intervals (Supplementary Table S7). Open symbols represent trait differences and filled symbols represent trait means. The relative importance of the variables are reported on the right side of the panels and can be interpreted as the probability that the variable appears in the best model. Hatched bars represent trait differences and filled bars represent trait means. Colours refer to the type of traits, with above- and below-ground traits represented in green and brown, respectively. Adjusted *R*^2^ values averaged across the top 10 models ($\bar{R_{adj}^{2}}$) are also reported.

RYTs were highly variable in both treatments (Fig. 3). The trait composition of the mixtures poorly explained RYT variability in total biomass observed in the R+ treatment (Fig. 5A, average adjusted *R*^2^ over the top 10 models=0.10). In contrast, trait composition explained up to 49% of RYT variation in the R– treatment (Fig. 5B). Most of this variability was explained by the average root area of the two varieties grown in mixture (*R*^2^=47% in a model with average root area as the single explanatory variable), which had a negative effect on RYT (Fig. 5B; Supplementary Table S7): mixing two genotypes with higher average root area in pure stands resulted in a decrease in biomass production in the mixture. We obtained similar results when performing the analysis on shoot biomass alone (Supplementary Fig. S2A, B; Supplementary Table S7). Traits had higher explanatory power on root biomass RYT in the R+ treatment (Supplementary Fig. S2C; average adjusted *R*^2^ over the top 10 models=0.34), with strong negative effects of average leaf number and average root area. In the R– treatment, however, as for the other biomass components, average root area was the main explanatory trait with a negative effect on RYT (Supplementary Fig. S2D).

The negative relationship between average root area and RYT was mostly driven by a negative relationship between average root area and complementarity effects in R–: mixing two varieties with high root areas in pure stands reduced biomass production for both varieties in the mixture (Supplementary Fig. S3A, B; Supplementary Table S7). In R–, the selection effect was mostly explained by the difference in root area between the varieties: the variety with the highest biomass production in pure stands tended to lose more biomass in mixture when it was grown with a variety that had a very different root area in pure stands. (Supplementary Fig. S3C, D; Supplementary Table S8). In R+, traits had much weaker explanatory power: we found no significant association between traits and complementarity effects (Supplementary Fig. S3A). Selection effects were mostly driven by a combined positive effect of the average root length and a negative effect of the average root area (*R*^2^=27%, Supplementary Fig. S3C).

### Relationship between root area and mixture biomass

Interpreting seedling biomass as a proxy for plant performance and final yield, one might conclude from our results that there was a global negative interaction between varieties when grown in mixtures under limiting conditions, and that varieties with higher root areas in pure stands had more negative interactions in mixtures; for example, they were more competitive with each other than varieties with low root areas, resulting in a negative complementarity effect. Following this line of reasoning, it may initially seem puzzling why such varieties were not also highly competitive with themselves in pure stands: by construction, varieties with the highest root area are more likely to be paired with neighbours with lower rather than higher root area than themselves in mixtures, and thus to experience reduced below-ground competition in mixed stands. Another interpretation is that varieties with high root area in pure stands could be highly competitive varieties that reach such trait values through root proliferation in response to their neighbour in pure stands, which would itself be very competitive. Such strong investment into competition would in turn result in an overinvestment in biomass to outgrow the neighbour. As those varieties would be more likely to be paired with less competitive neighbours in mixtures, they would experience a relaxed competition and thus disengage from the competitive ‘arms race’, resulting in lower root area and lower biomass. If the root area of the most competitive variety goes below the root area of the less competitive variety, this would lead to a synergistic effect where the less competitive variety also disengages from the arms race in a mixture.

**Fig. 6.**
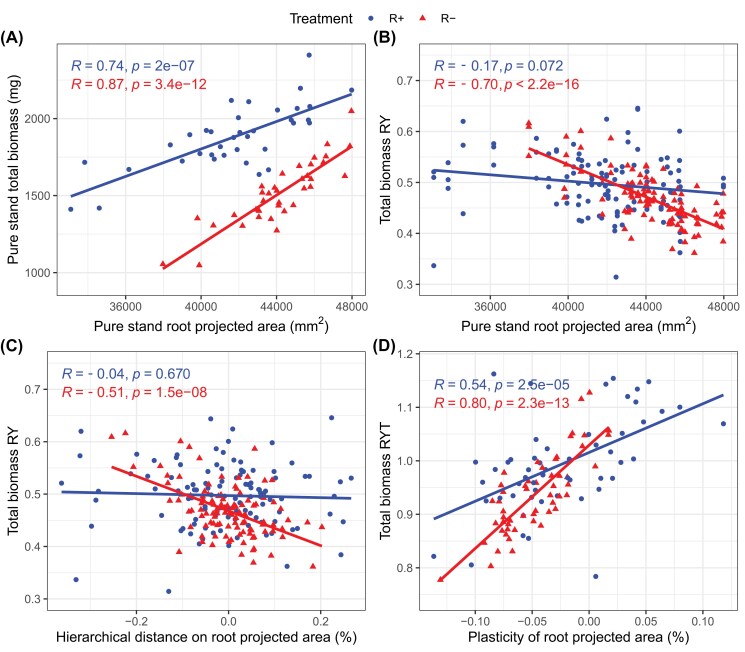
Interactions between root area, resource availability, and biomass. (A) Relationship between the total biomass of the pure stands and their root area (*n*=36 per treatment). (B) Relationship between RY computed on total biomass and root area measured in pure stands (*n*=108 per treatment). (C) Relationship between RY computed on total biomass and the hierarchical distance on root area; that is; the difference between the root area of the focal and the root area of the neighbour, both measured in pure stands (*n*=108 per treatment). (D) Relationship between RYT computed on total biomass and root area plasticity; that is; the difference between the expected (based on pure stands) and the observed root area (*n*=54 per treatment). Pearson correlation coefficients (*R*) and *P-*values refer to simple linear models fitted independently in the R+ (blue, circle) and R– (red, triangles) treatments.

We tested this hypothesis by examining the relationships between the traits of the varieties in pure stands and their individual biomass and biomass responses to mixture cultivation. We found that there was a strong positive relationship between the total biomass of the pure stands and their root area (Fig. 6A). This relationship was stronger in the R– treatment (Fig. 6A; Pearson’s *R*=0.87, slope=0.079 mg mm^–2^, *P*<0.001) than in the R+ treatment (Pearson’s *R*=0.74, slope=0.0446 mg mm^–2^, *P*<0.001). Consistent with our hypothesis, varieties with the highest root areas in pure stands and the highest hierarchical distances in root area with their neighbours were the ones with the strongest biomass reduction in mixed stands in the R– treatment (Fig. 6B, Pearson’s *R*= –0.70, *P*<0.001; Fig. 6C, Pearson’s *R*= –0.51, *P*<0.001). In contrast, varieties which had lower root areas than their neighbours (i.e. negative hierarchical distance from their neighbour in Fig. 6C) produced more biomass in mixtures than in pure stands (RY >0.5). This effect was not symmetrical: for a similar hierarchical distance, varieties which were lower in the hierarchy gained less biomass than the biomass loss observed for varieties placed higher in the hierarchy (e.g. at hierarchical distance= –0.2, estimated RY=0.53, while at hierarchical distance=0.2, estimated RY=0.40, Fig. 6C). Also, when the hierarchical distance was negative but close to 0 (greater than –0.1), the varieties still produced less biomass in mixture than in pure stands. None of these relationships was significant in the R+ treatment (Fig. 6B, C). Finally, the strongest biomass reductions occurred in mixtures where the observed root area was lower than the root area predicted from the pure stands, namely where phenotypic plasticity led to reduced root area (Fig. 6D). Again, the relationship between RYT and root area plasticity was stronger in the R– treatment (Pearson’s *R*=0.80, *P*<0.001, slope=1.93%^–1^) than in the R+ treatment (Pearson’s *R*=0.54, *P*<0.001, slope=0.91%^–1^). We obtained identical results when carrying out these analyses on shoot and root biomass separately, except that the differences between the R+ and R– treatments were less marked for root biomass (Supplementary Figs S4, S5). Altogether, these results support the idea that low RYTs in the R– treatment resulted from relaxed below-ground competition in mixed relative to pure stands.

## Discussion

### Shift in root allocation under water and nutrient limitation

Wheat seedling growth was strongly affected by the limitation of water and nutrients in our experiment. Resource limitation triggered an overall reduction in biomass, along with a shift in biomass allocation from the above-ground to the below-ground compartment (Fig. 2). This result is in line with the optimal allocation theory, which states that plants prioritize allocation to increase their uptake of the most limiting resources (Bloom *et al.*, 1985; Weiner, 2004). In our case, the high allocation to roots suggests that below-ground resource limitations were much stronger than above-ground resource limitations, a pattern consistent with what we intended to induce with our experimental protocol. In wheat, plasticity of the root-to-shoot ratio and high reallocation to roots have been shown to be advantageous under drought stress (Bacher *et al.*, 2021, 2022). Allocating carbon to the roots enhances access to water through deeper, longer, and more branched roots, which in turns helps maintain high stomatal conductance and physiological activity. We can thus hypothesize that higher root allocation in response to water and nutrient limitation reflected an adaptive response of the varieties.

### Biomass reduction in mixed relative to pure stands reflects a relaxation of competition

On average, mixture biomass was not significantly different from pure stand biomass under optimal growth conditions, indicating that there was no significant effect of interactions between varieties (Fig. 3). However, when water and nitrogen were limiting, mixtures produced significantly less biomass than expected from their pure stand components (Fig. 3), which was mainly explained by a negative complementarity effect and, to a lesser extent, by a negative selection effect (Fig. 4). At first glance, and if we interpret seedling biomass as a good proxy for final yield, this result suggests that negative interactions prevailed in our experiment, contradicting ecological theories and numerous experimental and observational studies where plant diversity effects are generally found to be more positive under harsher environments (Bertness and Callaway, 1994; Craven *et al.*, 2016). Such strong negative complementarity effects could be consistent with kin discrimination, whereby groups of genetically related individuals outperform groups of unrelated individuals. However, kin discrimination has never been reported in wheat, and its existence in plants is still debated (Pennisi, 2019; Anten and Chen, 2021). A more parsimonious interpretation is that water and nutrient limitation triggered an arms race between plants of highly competitive varieties in pure stands, which manifested through root proliferation and ultimately overinvestment in both below- and above-ground biomass. According to previous studies on root responses to the presence of competitor plants, such proliferation could have been triggered either by a direct perception of the presence of a competitive neighbour through the detection of chemical cues in the rhizosphere, or by the perception of resource depletion caused by the neighbour (Schenk, 2006; Pierik *et al.*, 2013). Such highly competitive varieties experienced reduced competition in mixed stands and thus disengaged from this competitive arms race and produced less biomass. When the difference in root area between two varieties was relatively small and they both had high root areas in pure stands (resulting in high average root area and small root area differences), the relaxation of competition was synergistic: the reduction in root area of the most competitive variety led to a lower root area than that of the neighbour in pure stands, leading to a simultaneous decrease in competition intensity for the neighbour, and finally to a negative complementarity effect. In our case, we interpreted such a ‘negative’ complementarity effect as a reduction of competition intensity which translated into a reduction of biomass. This interpretation is based on the hypothesis that the biomass of a 3-week-old wheat seedling in a stand is a better indicator of the competitiveness of the plant than of its future grain production. In cereals such as wheat, early seedling growth and vigour are traditionally targeted by plant breeders as favourable traits for competitiveness against weeds (Lemerle *et al.*, 1996; Bertholdsson, 2005; Hendriks *et al.*, 2022). However, in the absence of weeds and under limited resource conditions, intra-specific plant–plant interactions can become a strong determinant of yield per unit area. In this context, favouring competitive seedlings might actually decrease yields because the competitiveness of a seedling would essentially be directed towards itself. Supporting this hypothesis, Colombo *et al.* (2022) found that seedling biomass measured on the same phenotyping platform was negatively correlated with grain yields using a large dataset of 715 bread wheat varieties and 200 durum wheat varieties grown in 42 contrasting environments.

### No evidence for niche complementarity driven by trait differences between seedlings

While we found strong complementarity effects between varieties under resource limitation, these effects were mostly driven by average trait values in pure stands, and we found no evidence that trait differences between varieties contributed to complementarity effects (Fig. 5). This could be explained by the fact that functional differences between varieties were not large enough 3 weeks after sowing to generate complementarity effects. A second hypothesis is that we did not measure the ‘right’ traits to detect complementarity effects, notably below-ground. Our phenotyping method did not allow us to access traits such as root diameter or root tissue density. Other traits could not be computed due to root overlapping between adjacent plants (e.g. root angle) or to the reduced size of the RhizoTube® (e.g. root depth). However, such traits have rarely been found to be associated with complementarity effects in ecological studies (Fort *et al.*, 2014; Bakker *et al.*, 2018). Previous studies in rice varietal mixtures also failed to find evidence of below-ground niche complementarity driven by differences in morphological and architectural root traits such as rooting depth, root diameter, or specific root length (Montazeaud *et al.*, 2018). Overall, this study confirms that root trait differences alone are less likely to generate complementarity effects in varietal mixtures than they are, for example, in intercropping where differences in root foraging strategies between species can be more significant (Zhang *et al.*, 2014; Homulle *et al.*, 2022; Schmutz and Schöb, 2023).

### Root area as the main driver of competitive hierarchy

We identified root area as the main driver of plant biomass and mixing effects in our experiment (Figs 5, 6). The average root area of the two varieties in pure stands could explain up to 50% of RYT variability under resource-limiting conditions. A higher root area in pure stands was associated with a greater biomass reduction in mixture, especially when the mixture partner had a lower root area. These results support the idea of an early-stage below-ground competitive hierarchy between varieties (Kunstler *et al.*, 2012), where higher root area is associated with higher competitive ability. In line with these results, root functional traits associated with root foraging and absorption potential have already been shown to shape early-stage competitive hierarchies between grassland species (Fort *et al.*, 2014; Ravenek *et al.*, 2016; Wagg *et al.*, 2017). The very high explanatory power of root area found in our study might be explained by the fact that this trait integrates several functional dimensions of the root system that are classically captured by distinct traits (e.g. root length density, specific root length, or root diameter).

### Root plasticity contributes to mixing effects

We found that root area in mixed stands deviated from root area observed in pure stands, indicating a plastic response of the root system to neighbour genotype identity (Fig. 6D). On average, root area decreased in mixed relative to pure stands, and the stronger the decrease, the lower the RYT, especially in the R– treatment. In accordance with ecological theory, our results thus support the view that phenotypic plasticity contributes to mitigate competition intensity in diverse plant communities (Callaway *et al.*, 2003; Schiffers *et al.*, 2011; Burns and Strauss, 2012). In varietal mixtures, previous studies have already shown that plasticity in above-ground traits such as plant height, specific leaf area, or canopy cover contribute to overyielding, notably by increasing trait divergence between varieties (Kong and Zhao, 2023; Tschurr *et al.*, 2023; Su *et al.*, 2024). We here show that plastic root changes may contribute to mixture performance by decreasing biomass production at the seedling stage, which could ultimately translate into higher yields through reduced early stage competition between seedlings. We also show that plasticity contributes to mixture performance not only by increasing phenotypic differences between varieties, but also by displacing the average trait value of the varieties.

### Practical implications for plant breeding

Crop performance, including grain yield, is affected by trade-offs between individual competitiveness and group performance. As pointed out by plant breeders several years ago (Donald, 1968), and more recently revisited with the concepts of Darwinian Agriculture, or Evolutionary Agroecology, these trade-offs can hold promising opportunities to increase productivity, notably by reversing past selection for individual competitiveness (Denison *et al.*, 2003; Weiner, 2019). This can be achieved either by selecting on group performance (i.e. selecting on population yield) (Montazeaud *et al.*, 2020), or by targeting key traits related to competitive ability. Our study suggests that selecting genotypes with reduced root area in pure stands and reduced root area proliferation in the presence of neighbours could be a way to reduce competition intensity between seedlings, which could then translate into higher yields. This result aligns with classical prediction of game theory models: investment in root biomass beyond the payoff point where soil resource absorption compensates the cost of the root system only happens at cost for neighbouring plants, leading to a TOC (Hardin, 1968; Gersani *et al.*, 2001; Anten and Vermeulen, 2016). Interestingly, modern wheat varieties that have been subject to selection for increased population yield for many generations display reduced root biomass at the seedling stage (Colombo *et al.*, 2022), and reduced root proliferation in the presence of a neighbour compared with old landraces (Zhu *et al.*, 2019). In oat and barley, excessive growth of the root system in the topsoil is also associated with yield reductions (Vain *et al.*, 2023).

Beyond direct selection on root traits in pure stand varieties, which could be challenging to implement, mixing varieties could be another solution to resolve below-ground TOCs. This solution would allow benefit to be obtained from other known positive effects of genetic diversity at the same time (e.g. improved disease control, Wolfe, 1985; Mundt *et al.*, 1995; Zhu *et al.*, 2000; Finckh and Wolfe, 2006). Our study suggests that highly competitive varieties could benefit from relaxed below-ground competition in varietal mixtures, which could translate into overyielding. Conveniently, the choice of the varieties could be guided by their root projected area measured in pure stands. Of course, the effect of relaxed competition at the seedling stage on final grain yield would still need to be validated in field trials. Yet, in our particular experimental set-up, previous studies have already shown that the biomass of the seedling measured in the phenotyping platform after 3 weeks of growth was negatively correlated with grain yields measured on the same varieties in the field (Colombo *et al.*, 2022), suggesting that higher seedling biomass is indeed detrimental for final yields in comparable conditions. This means that promoting phenotypic differences between varieties is not the only way to reduce competition and may only work for some traits, such as phenological traits (Fletcher *et al.*, 2019; Lowry *et al.*, 2020) or traits related to light interception (Su *et al.*, 2024).

## Supplementary data

The following supplementary data are available at *JXB* online.

Table S1. List of the 36 wheat varieties used in the experiment.

Table S2. List of the 54 binary mixtures used in the experiment.

Table S3. Description of the nutrient solution.

Table S4. ANOVA of biomass components and traits in pure stands to test the treatment effect.

Table S5. ANOVA to test the effect of the treatment on RYTs.

Table S6. ANOVA to test the effect of the treatment on complementarity effects (CEs) and selection effects (SEs).

Table S7. Ten best fitting models between RYTs on above-ground, below-ground, and total biomass and mixture trait composition.

Table S8. Ten best fitting models between complementarity effects (CEs) and selection effects (SEs) measured on total biomass and mixture trait composition.

Fig. S1. RhizoTube® monitoring.

Fig. S2. Relationships between the trait composition of the mixtures and their above- and below-ground RYTs.

Fig. S3. Relationships between the trait composition of the mixtures and complementarity effects (CEs) and selection effects (SEs) measured on total biomass.

Fig. S4. Bivariate plots illustrating the interaction between root area, resource availability, and shoot biomass

Fig. S5. Bivariate plots illustrating the interaction between root area, resource availability, and root biomass.

eraf163_suppl_Supplementary_Tables_S1-S8_Figures_S1-S5

## Data Availability

All primary data to support the findings of this study are openly available in Zenodo at https://doi.org/10.5281/zenodo.14860837 (Montazeaud *et al.*, 2025).

## Abbreviations

CE: complementarity effect
RY: relative yield
RYT: relative yield total
SE: selection effect
