# Supplementary material for: Mixing varieties mitigates early root competition in wheat under water and nutrient limitation
Source: J Exp Bot. 2025 Apr 26;76(14):4171–84. doi: 10.1093/jxb/eraf163 (PMC12448814; doi:10.1093/jxb/eraf163)
Supplement: eraf163_suppl_Supplementary_Tables_S1-S8_Figures_S1-S5 [file eraf163_suppl_supplementary_tables_s1-s8_figures_s1-s5.pdf]

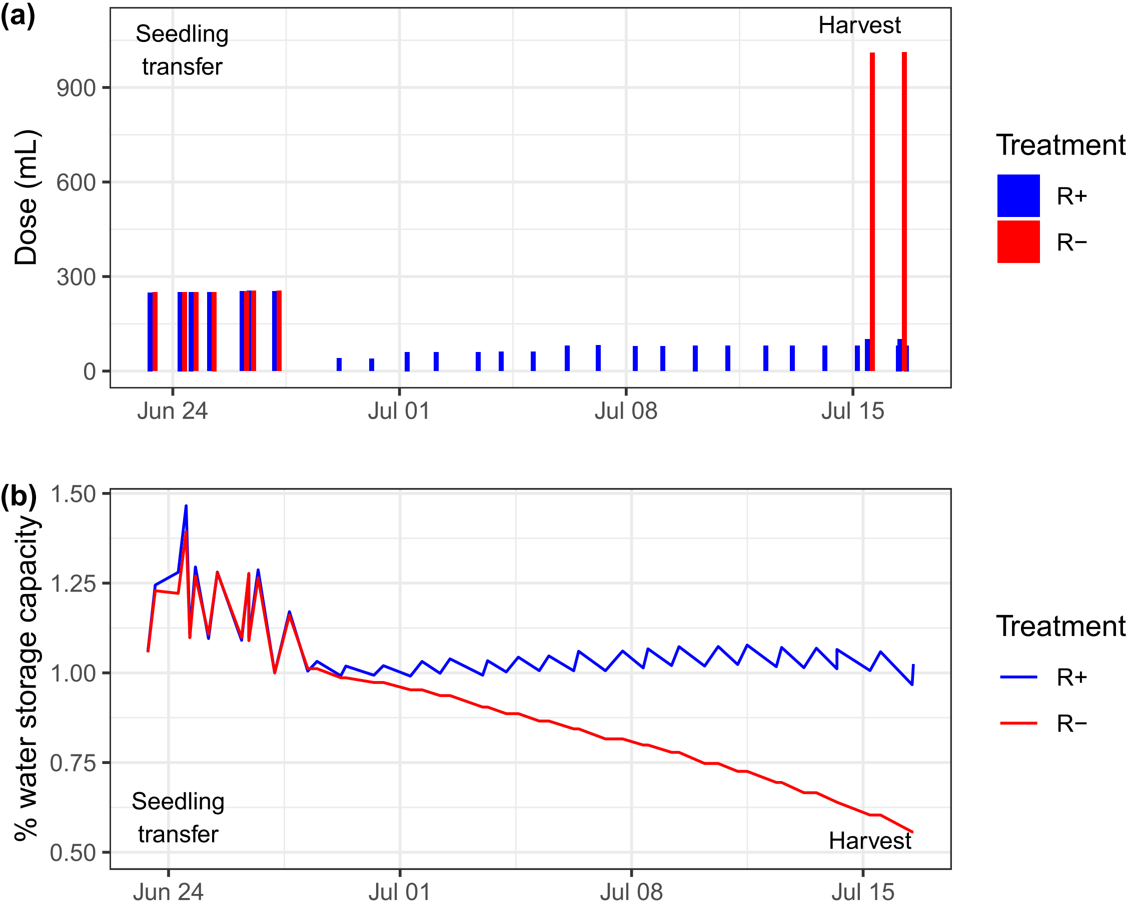

Supplementary Figure 1: RhizoTubes® monitoring. Measurements of nutrient solution inputs (**a**) and water status (**b**) of the RhizoTubes® in the R+ treatment (blue) and the R- treatment (red) over the course of the experiment. Values are averaged over all RhizoTubes®. Seedlings were transferred in the RhizoTubes on the 24th of June, and plants were harvested from the 16th to the 19th of July 2019.

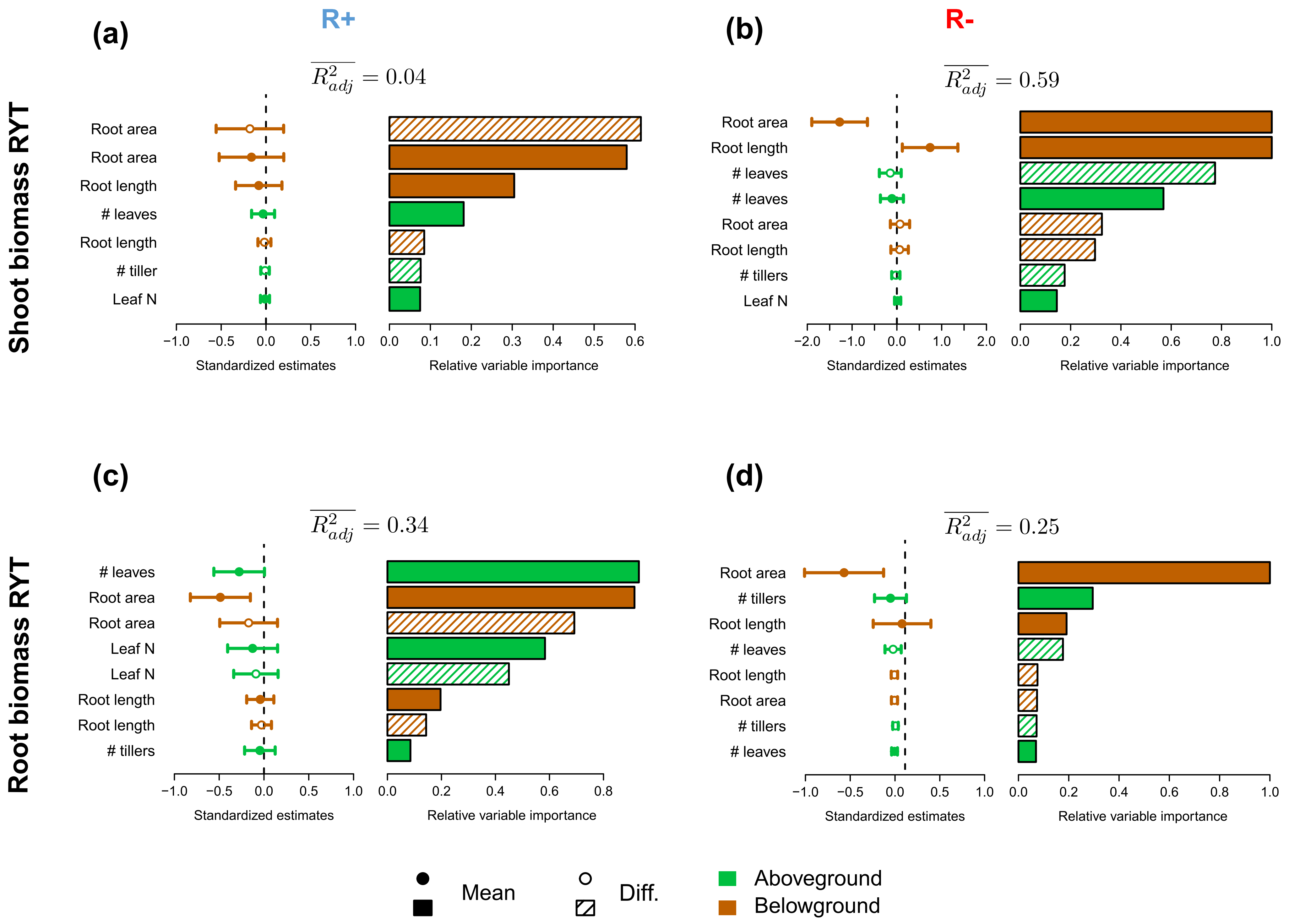

Supplementary Figure 2: Relationships between the trait composition of the mixtures and their above and belowground RYTs. Standardized effects of traits on mixture RYTs measured on above- (**a** and **b**) and below- (**c** and **d**) ground biomass in the R+ treatment (**a** and **c**) and R- treatment (**b** and **d**). Backward model selection was performed on a full model with RYT as the response variable and all trait means and differences as explanatory variables. Based on AIC_C, the top-ten models were retained to compute model-averaged estimates reported on the left side of the panels with their 95% unconditional confidence intervals (Supplementary Table 7). Empty symbols represent trait differences and filled symbols represent trait means. The relative importance of the variables are reported on the right side of the panels and can be interpreted as the probability that the variable appears in the best model. Hatched bars represent trait differences and filled bars represent trait means. Colours refer to the type of traits, with aboveground traits and belowground traits represented in green and brown, respectively. Adjusted R-squared averaged across the top-ten models ($\overline{R^2_{adj}}$) are also reported.

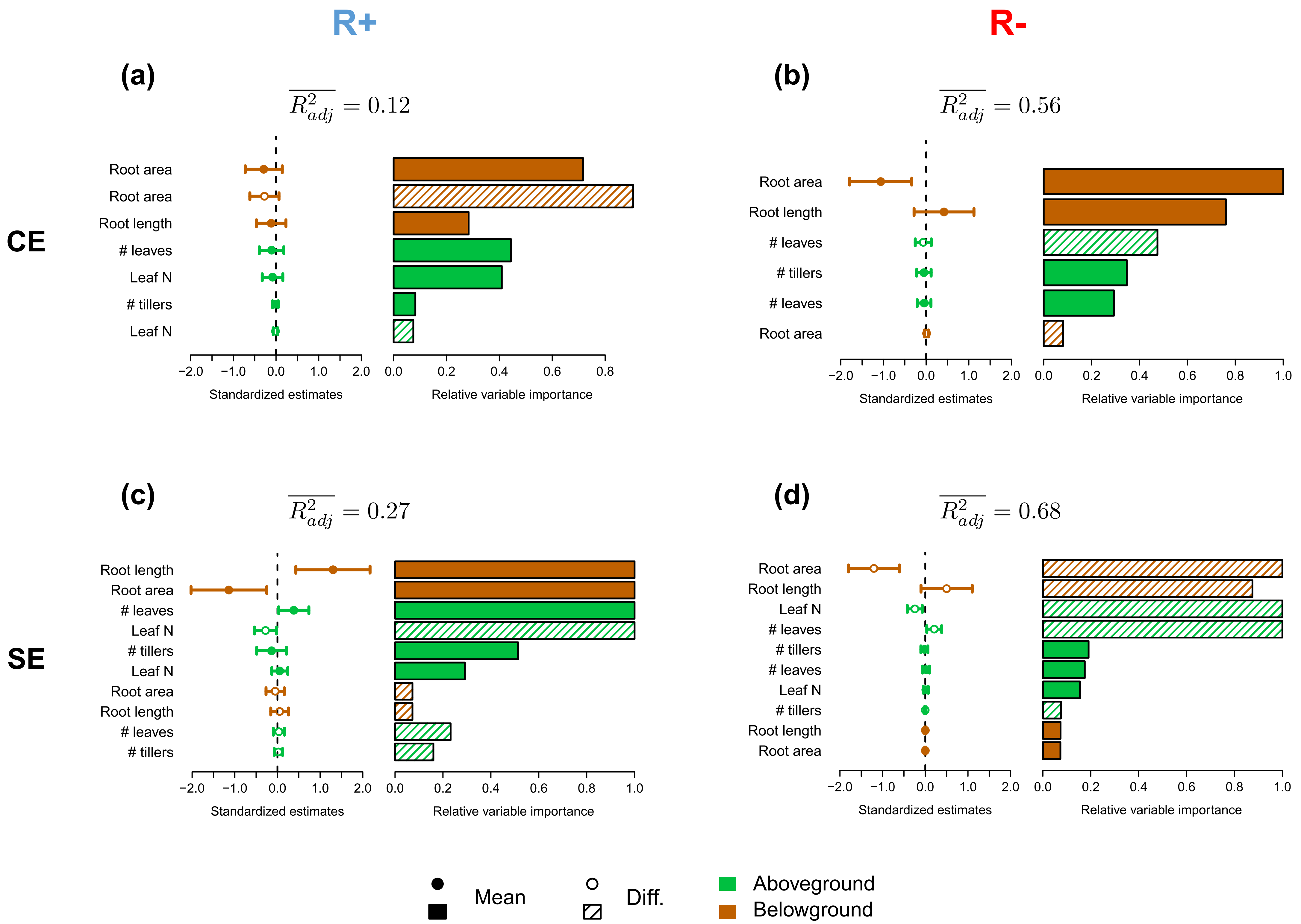

Supplementary Figure 3: Relationships between the trait composition of the mixtures and complementarity and selection effects (CE and SE, respectively). Standardized effects of traits on mixture CE (**a** and **b**) and SE (**c** and **d**) computed on total biomass in the R+ treatment (**a** and **c**) and R- treatment (**b** and **d**). Backward model selection was performed on a full model with CE (or SE) as the response variable and all trait means and differences as explanatory variables. Based on AIC_C, the top-ten models were retained to compute model-averaged estimates reported on the left side of the panels with their 95% unconditional confidence intervals (Supplementary Table 8). Empty symbols represent trait differences and filled symbols represent trait means. The relative importance of the variables are reported on the right side of the panels and can be interpreted as the probability that the variable appears in the best model. Hatched bars represent trait differences and filled bars represent trait means. Colours refer to the type of traits, with aboveground traits and belowground traits represented in green and brown, respectively. Adjusted R-squared averaged across the top-ten models ($\overline{R^2_{adj}}$) are also reported.

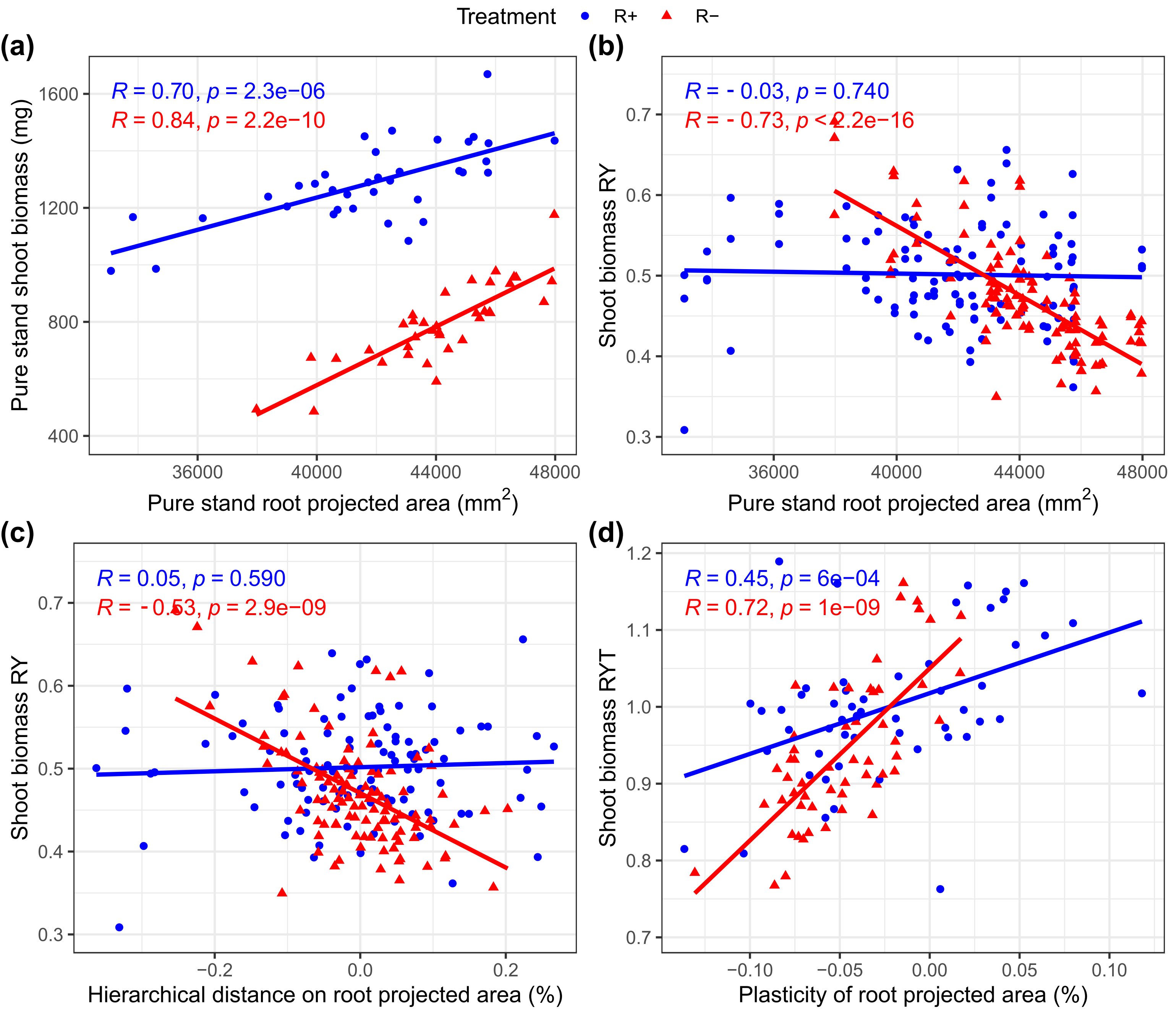

Supplementary Figure 4: Interactions between root area, resource availability, and shoot biomass. (**a**) relationship between the shoot biomass of the pure stands and their root area (n = 36 per treatment), (**b**) relationship between RY computed on shoot biomass and root area measured in pure stands (n = 108 per treatment), (**c**) relationship between RY computed on shoot biomass and the hierarchical distance on root area, i.e., the difference between the root area of the focal and the root area of the neighbour, both measured in pure stands (n = 108 per treatment), (**d**) relationship between RYT computed on shoot biomass and root area plasticity, i.e., the difference between the expected (based on pure stands) and the observed root area (n = 54 per treatment). Pearson correlation coefficients (*R*) and p-values (*p*) refer to simple linear models fitted independently in the R+ (blue, circle) and R- (red, triangles) treatments.

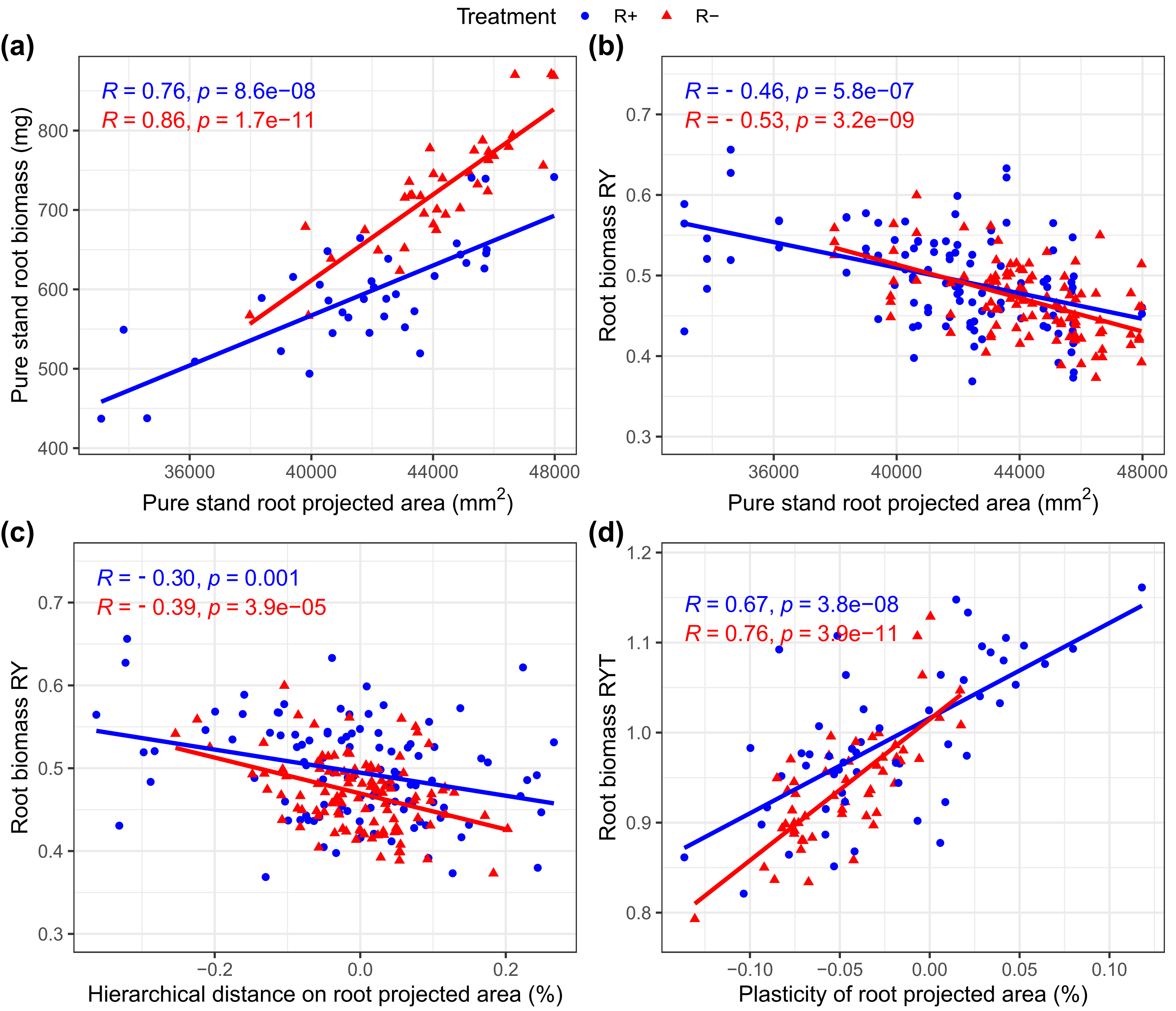

Supplementary Figure 5: Interactions between root area, resource availability, and root biomass. (**a**) relationship between the root biomass of the pure stands and their root area (n = 36 per treatment), (**b**) relationship between RY computed on root biomass and root area measured in pure stands (n = 108 per treatment), (**c**) relationship between RY computed on root biomass and the hierarchical distance on root area, i.e., the difference between the root area of the focal and the root area of the neighbour, both measured in pure stands (n = 108 per treatment), (**d**) relationship between RYT computed on root biomass and root area plasticity, i.e. the difference between the expected (based on pure stands) and the observed root area (n = 54 per treatment). Pearson correlation coefficients (*R*) and p-values (*p*) refer to simple linear models fitted independently in the R+ (blue, circle) and R- (red, triangles) treatments.

Supplementary Table 1: Variety information.

Genotype	Panel	Collection
ACADUR	UNIBO	Bologna University
ANTALIS	CREA	CREA
ANVERGUR	GPDUR	Bologna University
ARDENTE	UNIBO	Bologna University
ASTERIX	CREA	CREA
AVENTUR	GPDUR	Arvalis
AZEGHAR-2_DP128	UNIBO	Bologna University
BALSAMO	CREA	CREA
BOLIDO-DP034	UNIBO	Bologna University
CASANOVA	CREA	CREA
CHAM-1_DP136	UNIBO	Bologna University
COLOSSEO_DP087	UNIBO	Bologna University
EL4X_120	EPO	INRAE Montpellier
EL4X_194	EPO	INRAE Montpellier
EL4X_316	EPO	INRAE Montpellier
EL4X_35	EPO	INRAE Montpellier
EL4X_428	EPO	INRAE Montpellier
EL4X_464	EPO	INRAE Montpellier
FURIO_CAMILLO	CREA	CREA
GIUSTO	CREA	CREA
KOFA	UNIBO	Bologna University
L2574	CREA	CREA
LAHAN	GPDUR	Arvalis
LGBORIS	GPDUR	Arvalis
LLOYD	UNIBO	Bologna University
MIKI-1_DP161	UNIBO	Bologna University
MONASTIR	GPDUR	Arvalis
MURANO	GPDUR	Arvalis
NEMESIS	GPDUR	Arvalis
NOBILIS	GPDUR	Arvalis
ORJAUNE	GPDUR	Arvalis
PLUSSUR	GPDUR	Arvalis
QUALIDOU	GPDUR	Arvalis
RAMIREZ	CREA	CREA
Selcuklu-97	CREA	CREA
SVEVO	CREA	CREA

Supplementary Table 2: List of the 54 binary mixtures.

Genotype 1	Genotype 2
ACADUR	ANVERGUR
ACADUR	ARDENTE
ASTERIX	EL4X_35
ASTERIX	MIKI-1_DP161
ASTERIX	NEMESIS
AVENTUR	EL4X_194
AVENTUR	ORJAUNE
AZEGHAR-2_DP128	KOFA
AZEGHAR-2_DP128	NEMESIS
BALSAMO	ARDENTE
BALSAMO	AVENTUR
BOLIDO-DP034	EL4X_194
COLOSSEO_DP087	AZEGHAR-2_DP128
COLOSSEO_DP087	KOFA
COLOSSEO_DP087	QUALIDOU
EL4X_120	CHAM-1_DP136
EL4X_120	SELCUKLU-97
EL4X_316	ORJAUNE
FURIO_CAMILLO	ACADUR
FURIO_CAMILLO	EL4X_428
FURIO_CAMILLO	PLUSSUR
GIUSTO	ANTALIS
GIUSTO	BOLIDO-DP034
GIUSTO	CASANOVA
L2574	CASANOVA
L2574	EL4X_316
L2574	QUALIDOU

Genotype 1	Genotype 2
LAHAN	BOLIDO-DP034
LAHAN	EL4X_120
LAHAN	EL4X_464
LGBORIS	EL4X_35
LGBORIS	NEMESIS
LLOYD	ANVERGUR
LLOYD	EL4X_464
MIKI-1_DP161	EL4X_464
MONASTIR	BALSAMO
MONASTIR	CHAM-1_DP136
MONASTIR	EL4X_35
MURANO	ANTALIS
MURANO	QUALIDOU
MURANO	SVEVO
NOBILIS	EL4X_316
NOBILIS	EL4X_428
NOBILIS	LLOYD
ORJAUNE	KOFA
PLUSSUR	CHAM-1_DP136
PLUSSUR	MIKI-1_DP161
RAMIREZ	ANTALIS
RAMIREZ	CASANOVA
RAMIREZ	LGBORIS
SELCUKLU-97	ANVERGUR
SELCUKLU-97	EL4X_194
SVEVO	ARDENTE
SVEVO	EL4X_428

Supplementary Table 3: Description of the nutrient solution.

Macronutrients			
**molecule**	**molar mass**	**concentration Mm/L**	**concentration g/L**
K2HPO4	174,18	1	0,17418
KNO3	101,1	5	0,5055
Ca(NO3)2+4H2O	236,15	2,5	0,590375
MgSO4+7H2O	246	2	0,492
CaCl2 +2H2O	147,01	2	0,29402
Micronutrients			
**molecule**	**molar mass**	**concentration µM/L**	**concentration mg/L**
H3Bo3	61,83	10	0,6183
MnCl2+4H2O	197,91	4,5	0,890595
ZnCl2	138	0,7	0,0966
Mo(Na)2 O4	241,95	0,2	0,04839
CuCl2+2H2O	170,48	0,4	0,068192
Fe EDTA	367	56	20,552

Supplementary Table 4: Analysis of Variance (ANOVA) of biomass components and traits in pure stands. Pure stand data was summed per Rhizotube® (except leaf N which was averaged per Rhizotube®). We used Type III analysis of Variance using the Kenward-Roger's method on mixed models where the identity of the variety was used as a random effect on both the intercept and the slope of the treatment effect (R+ vs R- treatment). For each fixed effects, we report the sum of squares ("Sum Sq"), the mean squares ("Mean Sq"), the numerator degrees of freedom ("NumDF"), the denominator degrees of freedom ("DenDF"), the value of the *F* statistic ("F value"), and the *p*-value ("Pr(>F)").

# leaves on the main stem						
	Sum Sq	Mean Sq	NumDF	DenDF	F value	Pr(>F)
Sampling_date	339,49	339,49	1,00	195,21	47,15	< 0,001
Block	14,50	7,25	2,00	141,13	1,01	0,3680
Treatment	318,11	318,11	1,00	35,21	44,18	< 0,001

# tillers						
	Sum Sq	Mean Sq	NumDF	DenDF	F value	Pr(>F)
Sampling_date	85,99	85,99	1,00	186,25	13,34	0,0003
Block	11,65	5,82	2,00	141,12	0,90	0,4073
Treatment	1930,77	1930,77	1,00	35,22	299,63	< 0,001

Leaf N (%)						
	Sum Sq	Mean Sq	NumDF	DenDF	F value	Pr(>F)
Sampling_date	0,00	0,00	1,00	173,63	0,08	0,7790
Block	0,67	0,33	2,00	129,90	17,33	< 0,001
Treatment	3,60	3,60	1,00	35,18	187,43	< 0,001

Shoot biomass (mg)						
	Sum Sq	Mean Sq	NumDF	DenDF	F value	Pr(>F)
Sampling_date	2435423,50	2435423,50	1,00	195,00	41,09	< 0,001
Block	93148,74	46574,37	2,00	141,13	0,79	0,4577
Treatment	10295313,57	10295313,57	1,00	35,20	173,71	< 0,001

Root biomass (mg)						
	Sum Sq	Mean Sq	NumDF	DenDF	F value	Pr(>F)
Sampling_date	583869,24	583869,24	1,00	197,39	25,65	< 0,001
Block	232598,28	116299,14	2,00	141,15	5,11	0,0072
Treatment	880216,28	880216,28	1,00	35,23	38,67	< 0,001

Total biomass (mg)						
	Sum Sq	Mean Sq	NumDF	DenDF	F value	Pr(>F)
Sampling_date	5495123,01	5495123,01	1,00	195,91	38,44	< 0,001
Block	562901,40	281450,70	2,00	141,14	1,97	0,1434
Treatment	6245857,09	6245857,09	1,00	35,22	43,69	< 0,001

Supplementary Table 4 continued

Root:Shoot ratio						
	Sum Sq	Mean Sq	NumDF	DenDF	F value	Pr(>F)
Sampling_date	0,01	0,01	1,00	191,46	2,65	0,1049
Block	0,08	0,04	2,00	141,15	11,25	< 0,001
Treatment	5,93	5,93	1,00	35,22	1629,48	< 0,001

Root length (mm)						
	Sum Sq	Mean Sq	NumDF	DenDF	F value	Pr(>F)
Block	318426545,36	159213272,68	2,00	139,52	3,96	0,0213
Treatment	555238364,10	555238364,10	1,00	34,94	13,81	0,0007

Root projected area (mm²)						
	Sum Sq	Mean Sq	NumDF	DenDF	F value	Pr(>F)
Block	170499632,38	85249816,19	2,00	142,00	2,29	0,1047
Treatment	229762346,26	229762346,26	1,00	35,00	6,18	0,0179

Supplementary Table 5: Analysis of Variance (ANOVA) of RYTs. Type III analysis of Variance using the Kenward-Roger's method on mixed models where the identity of the genotypic pair (concatenation of the identity of the two genotypes in a RhizoTube®) was used as a random effect on the intercept. We report the fixed effect of the treatment with the sum of squares ("Sum Sq"), the mean squares ("Mean Sq"), the numerator degrees of freedom ("NumDF"), the denominator degrees of freedom ("DenDF"), the value of the *F* statistic ("F value"), and the *p*-value ("Pr(>F)").

**RYT Shoot biomass (mg)**						
	Sum Sq	Mean Sq	NumDF	DenDF	F value	Pr(>F)
Treatment	0,08	0,08	1,00	53,00	26,23	< 0,001

**RYT Root biomass (mg)**						
	Sum Sq	Mean Sq	NumDF	DenDF	F value	Pr(>F)
Treatment	0,07	0,07	1,00	53,00	32,56	< 0,001

**RYT Total biomass (mg)**						
	Sum Sq	Mean Sq	NumDF	DenDF	F value	Pr(>F)
Treatment	0,09	0,09	1,00	53,00	34,87	< 0,001

Supplementary Table 6: Analysis of Variance (ANOVA) of complementarity and selection effects (CE and SE, respectively). Type III analysis of Variance using the Kenward-Roger's method on mixed models where the identity of the genotypic pair (concatenation of the identity of the two genotypes in a RhizoTube®) was used as a random effect on the intercept. We report the fixed effect of the treatment with the sum of squares ("Sum Sq"), the mean squares ("Mean Sq"), the numerator degrees of freedom ("NumDF"), the denominator degrees of freedom ("DenDF"), the value of the *F* statistic ("F value"), and the *p*-value ("Pr(>F)").

**CE Shoot biomass (mg)**						
	Sum Sq	Mean Sq	NumDF	DenDF	F value	Pr(>F)
Treatment	66878,00	66878,00	1,00	53,00	15,32	< 0,001

**CE Root biomass (mg)**						
	Sum Sq	Mean Sq	NumDF	DenDF	F value	Pr(>F)
Treatment	38347,00	38347,00	1,00	53,00	45,77	< 0,001

**CE Total biomass (mg)**						
	Sum Sq	Mean Sq	NumDF	DenDF	F value	Pr(>F)
Treatment	210974,00	210974,00	1,00	53,00	25,11	< 0,001

**SE Shoot biomass (mg)**						
	Sum Sq	Mean Sq	NumDF	DenDF	F value	Pr(>F)
Treatment	2148,20	2148,20	1,00	53,00	21,00	< 0,001

**SE Root biomass (mg)**						
	Sum Sq	Mean Sq	NumDF	DenDF	F value	Pr(>F)
Treatment	4,93	4,93	1,00	53,00	0,92	0,3406

**SE Total biomass (mg)**						
	Sum Sq	Mean Sq	NumDF	DenDF	F value	Pr(>F)
Treatment	1488,10	1488,10	1,00	53,00	14,62	< 0,001

Supplementary Table 7: Ten best fitting models between RYT on aboveground, belowground, and total biomass and mixture trait composition. The top-ten models are ranked according to their AICc. ΔAICc, model weights (“weight”), and adjusted R-squared (“R2_adj”) are reported . The “avg” and “diff” suffixes refer to trait averages and trait differences, respectively.

**Shoot biomass RYT - R+**										
ΔAIC_C	weight	R2_adj	Root_proj_area_diff	Root_proj_area_avg	Root_length_avg	#_leaves_avg	Root_length_diff	leaf_N_avg	#_tillers_avg	
0,00	0,15	0,05	-0,26	-0,31	NA	NA	NA	NA	NA	
0,30	0,13	0,05	-0,27	NA	-0,31	NA	NA	NA	NA	
0,54	0,12	0,00	NA	NA	NA	NA	NA	NA	NA	
0,80	0,10	0,02	NA	-0,19	NA	NA	NA	NA	NA	
1,02	0,09	0,06	-0,32	-0,29	NA	-0,17	NA	NA	NA	
1,03	0,09	0,06	-0,34	NA	-0,29	-0,19	NA	NA	NA	
1,15	0,08	0,03	NA	-0,27	NA	NA	-0,20	NA	NA	
1,17	0,08	0,01	NA	NA	-0,17	NA	NA	NA	NA	
1,37	0,08	0,05	-0,28	-0,34	NA	NA	NA	-0,14	NA	
1,40	0,08	0,05	-0,31	-0,28	NA	NA	NA	NA	-0,15	
**Shoot biomass RYT - R-**										
ΔAIC_C	weight	R2_adj	Root_proj_area_avg	Root_length_avg	#_leaves_diff	#_leaves_avg	Root_proj_area_diff	Root_length_diff	#_tillers_avg	leaf_N_diff
0,00	0,14	0,58	-1,18	0,61	NA	-0,22	NA	NA	NA	NA
0,13	0,14	0,59	-1,44	0,89	-0,22	NA	0,23	NA	NA	NA
0,40	0,12	0,59	-1,38	0,82	-0,22	NA	NA	0,22	NA	NA
0,72	0,10	0,60	-1,29	0,79	-0,19	-0,15	0,19	NA	NA	NA
0,96	0,09	0,60	-1,24	0,74	-0,18	-0,15	NA	0,18	NA	NA
0,99	0,09	0,58	-1,17	0,60	-0,11	-0,20	NA	NA	NA	NA
0,99	0,09	0,60	-1,23	0,72	-0,22	NA	NA	0,21	-0,14	NA
1,00	0,09	0,60	-1,29	0,79	-0,22	NA	0,22	NA	-0,13	NA
1,17	0,08	0,58	-1,25	0,71	NA	-0,23	NA	NA	NA	0,11
1,62	0,06	0,59	-1,26	0,73	-0,13	-0,21	NA	NA	NA	0,13

Supplementary Table 7 continued

**Root biomass RYT - R+**											
ΔAIC_C	weight	R2_adj	#_leaves_avg	Root_proj_area_avg	Root_proj_area_diff	leaf_N_avg	leaf_N_diff	Root_length_diff	#_tillers_avg	Root_length_avg
0,00	0,18	0,35	-0,33	-0,56	-0,24	-0,23	NA	NA	NA	NA
0,99	0,11	0,34	-0,32	-0,53	NA	-0,22	NA	-0,20	NA	NA
1,01	0,11	0,32	-0,25	-0,47	NA	-0,21	NA	NA	NA	NA
1,04	0,11	0,34	-0,28	-0,53	-0,25	NA	-0,21	NA	NA	NA
1,19	0,10	0,36	-0,30	-0,56	-0,26	-0,18	-0,14	NA	NA	NA
1,54	0,09	0,33	-0,31	NA	-0,28	NA	-0,23	NA	NA	-0,54
1,56	0,08	0,33	-0,28	-0,51	NA	NA	-0,20	-0,22	NA	NA
1,81	0,07	0,35	-0,25	-0,55	-0,26	-0,24	NA	NA	-0,13	NA
1,97	0,07	0,33	NA	-0,52	-0,23	NA	-0,24	NA	-0,26	NA
1,99	0,07	0,31	-0,32	-0,51	-0,21	NA	NA	NA	NA	NA
**Root biomass RYT - R-**										
ΔAIC_C	weight	R2_adj	Root_proj_area_avg	#_tillers_avg	Root_length_avg	#_leaves_diff	Root_length_diff	Root_proj_area_diff	#_tillers_dist	#_leaves_avg
0,00	0,19	0,25	-0,52	NA	NA	NA	NA	NA	NA	NA
0,53	0,15	0,26	-0,44	-0,17	NA	NA	NA	NA	NA	NA
0,89	0,12	0,26	-0,94	NA	0,44	NA	NA	NA	NA	NA
1,37	0,10	0,25	-0,50	NA	NA	-0,12	NA	NA	NA	NA
1,76	0,08	0,26	-0,41	-0,18	NA	-0,13	NA	NA	NA	NA
1,87	0,08	0,24	-0,55	NA	NA	NA	-0,09	NA	NA	NA
1,91	0,07	0,24	-0,55	NA	NA	NA	NA	-0,08	NA	NA
1,96	0,07	0,24	-0,54	NA	NA	NA	NA	NA	0,08	NA
2,03	0,07	0,24	-0,47	NA	NA	NA	NA	NA	NA	-0,08
2,09	0,07	0,26	-0,78	-0,15	0,35	NA	NA	NA	NA	NA

Supplementary Table 7 continued

Total biomass RYT - R+									
ΔAIC_C	weight	R2_adj	Root_proj_area_diff	Root_proj_area_avg	#_leaves_avg	Root_length_avg	leaf_N_avg	#_tillers_avg	Root_length_diff
0,00	0,13	0,12	-0,30	-0,36	-0,23	NA	NA	NA	NA
0,24	0,12	0,11	-0,32	NA	-0,25	-0,36	NA	NA	NA
0,28	0,12	0,09	-0,22	-0,39	NA	NA	NA	NA	NA
0,28	0,12	0,07	NA	-0,29	NA	NA	NA	NA	NA
0,32	0,11	0,14	-0,33	-0,40	-0,24	NA	-0,19	NA	NA
0,85	0,09	0,10	-0,24	-0,43	NA	NA	-0,18	NA	NA
0,97	0,08	0,10	-0,28	-0,36	NA	NA	NA	-0,19	NA
1,02	0,08	0,08	-0,23	NA	NA	-0,38	NA	NA	NA
1,08	0,08	0,05	NA	NA	NA	-0,27	NA	NA	NA
1,09	0,08	0,10	NA	-0,33	-0,22	NA	NA	NA	-0,26
**Total biomass RYT – R-**									
ΔAIC_C	weight	R2_adj	Root_proj_area_avg	Root_length_avg	#_tillers_avg	#_leaves_diff	#_leaves_avg	Root_proj_area_diff	
0,00	0,17	0,49	-1,28	0,63	NA	NA	NA	NA	
0,51	0,13	0,50	-1,12	0,53	-0,15	NA	NA	NA	
0,61	0,12	0,50	-1,14	0,57	NA	NA	-0,16	NA	
0,98	0,10	0,48	-0,60	NA	-0,19	NA	NA	NA	
1,01	0,10	0,49	-1,25	0,61	NA	-0,11	NA	NA	
1,28	0,09	0,51	-1,08	0,51	-0,16	-0,13	NA	NA	
1,52	0,08	0,49	-0,57	NA	-0,20	-0,13	NA	NA	
1,64	0,07	0,48	-0,58	NA	NA	NA	-0,18	NA	
1,70	0,07	0,50	-1,32	0,76	NA	-0,18	NA	0,16	
1,76	0,07	0,46	-0,69	NA	NA	NA	NA	NA	

Supplementary Table 8: Ten best fitting models between CE and SE computed on total biomass and mixture trait composition. The top-ten models are ranked according to their AICc. ΔAICc, model weights (“weight”), and adjusted R-squared (“R2_adj”) are reported. The “avg” and “diff” suffixes refer to trait averages and trait differences, respectively.

**CE - R+**									
ΔAIC_C	weight	R2_adj	Root_proj_area_avg	Root_proj_area_dist	Root_length_avg	#_leaves_avg	leaf_N_avg	#_tillers_avg	leaf_N_diff
0,00	0,13	0,15	-0,43	-0,34	NA	-0,23	-0,21	NA	NA
0,28	0,11	0,12	-0,38	-0,31	NA	-0,22	NA	NA	NA
0,30	0,11	0,12	NA	-0,34	-0,39	-0,24	NA	NA	NA
0,34	0,11	0,10	-0,41	-0,23	NA	NA	NA	NA	NA
0,35	0,11	0,12	-0,45	-0,26	NA	NA	-0,20	NA	NA
0,66	0,09	0,07	-0,30	NA	NA	NA	NA	NA	NA
0,85	0,09	0,14	NA	-0,36	-0,41	-0,25	-0,17	NA	NA
0,86	0,09	0,09	NA	-0,25	-0,40	NA	NA	NA	NA
0,93	0,08	0,14	-0,42	-0,32	NA	NA	-0,21	-0,19	NA
1,13	0,07	0,11	-0,42	-0,27	NA	NA	NA	NA	-0,17
**CE – R-**									
ΔAIC_C	weight	R2_adj	Root_proj_area_avg	Root_length_avg	#_leaves_diff	#_tillers_avg	#_leaves_avg	Root_proj_area_dist	
0,00	0,15	0,55	-1,28	0,58	NA	NA	NA	NA	
0,25	0,13	0,56	-1,25	0,57	-0,13	NA	NA	NA	
0,30	0,13	0,56	-1,14	0,52	NA	NA	-0,16	NA	
0,89	0,10	0,57	-1,10	0,48	-0,14	-0,14	NA	NA	
0,92	0,09	0,55	-1,15	0,50	NA	-0,13	NA	NA	
1,13	0,08	0,56	-1,13	0,52	-0,12	NA	-0,14	NA	
1,14	0,08	0,55	-0,63	NA	-0,15	-0,18	NA	NA	
1,21	0,08	0,54	-0,63	NA	NA	NA	-0,18	NA	
1,23	0,08	0,56	-1,31	0,70	-0,19	NA	NA	0,14	
1,41	0,07	0,54	-0,66	NA	NA	-0,17	NA	NA	

Supplementary Table 8 continued

**SE - R+**												
ΔAIC_C	weight	R2_adj	Root_length_avg	Root_proj_area_avg	#_leaves_avg	leaf_N_diff	#_tillers_avg	leaf_N_avg	Root_proj_area_dist	Root_length_diff	#_leaves_diff	#_tillers_diff
0,00	0,18	0,28	1,34	-1,16	0,48	-0,27	-0,27	NA	NA	NA	NA	NA
0,45	0,14	0,25	1,39	-1,25	0,31	-0,26	NA	NA	NA	NA	NA	NA
0,79	0,12	0,29	1,15	-0,95	0,49	-0,34	-0,27	0,18	NA	NA	NA	NA
1,29	0,09	0,26	1,20	-1,04	0,32	-0,33	NA	0,18	NA	NA	NA	NA
1,41	0,09	0,26	1,36	-1,22	0,24	-0,24	NA	NA	NA	NA	NA	0,16
1,48	0,08	0,26	1,44	-1,30	0,28	-0,24	NA	NA	NA	NA	0,15	NA
1,67	0,08	0,28	1,23	-1,07	0,29	-0,32	NA	0,21	NA	NA	0,18	NA
1,79	0,07	0,30	1,19	-1,06	0,52	-0,29	-0,31	NA	-0,73	0,71	NA	NA
1,81	0,07	0,28	1,32	-1,15	0,40	-0,26	-0,24	NA	NA	NA	NA	0,12
1,88	0,07	0,28	1,38	-1,21	0,44	-0,26	-0,24	NA	NA	NA	0,11	NA
**SE - R-**												
ΔAIC_C	weight	R2_adj	Root_proj_area_dist	Root_length_diff	leaf_N_diff	#_leaves_diff	#_tillers_avg	#_leaves_avg	leaf_N_avg	#_tillers_diff	Root_length_avg	Root_proj_area_avg
0,00	0,27	0,68	-1,27	0,57	-0,24	0,21	NA	NA	NA	NA	NA	NA
1,91	0,10	0,68	-1,29	0,57	-0,24	0,22	-0,06	NA	NA	NA	NA	NA
1,96	0,10	0,68	-1,24	0,51	-0,24	0,21	NA	NA	0,07	NA	NA	NA
2,24	0,09	0,68	-1,26	0,59	-0,25	0,20	NA	0,05	NA	NA	NA	NA
2,29	0,09	0,69	-1,28	0,63	-0,27	0,17	-0,16	0,17	NA	NA	NA	NA
2,57	0,08	0,68	-1,28	0,57	-0,23	0,23	NA	NA	NA	-0,02	NA	NA
2,60	0,07	0,68	-1,26	0,57	-0,24	0,21	NA	NA	NA	NA	0,02	NA
2,62	0,07	0,68	-1,27	0,58	-0,24	0,21	NA	NA	NA	NA	NA	0,02
2,67	0,07	0,66	-0,73	NA	-0,26	0,23	NA	NA	NA	NA	NA	NA
3,25	0,05	0,66	-0,76	NA	-0,25	0,21	NA	NA	0,11	NA	NA	NA
